# Supplementary material for: Magnesium Transporter MgtA revealed as a Dimeric P-type ATPase
Source: bioRxiv. 2024 Feb 29:2024.02.28.582502. Preprint. [Version 1] doi: 10.1101/2024.02.28.582502 (PMC10925321; doi:10.1101/2024.02.28.582502)
Supplement: 1 [file NIHPP2024.02.28.582502v1-supplement-1.pdf]

bioRxiv preprint doi: https://doi.org/10.1101/2024.02.28.582502; this version posted March 1, 2024. The copyright holder for this preprint (which was not certified by peer review) is the author/funder. This article is a US Government work. It is not subject to copyright under 17 USC 105 and is also made available for use under a CC0 license.

Supplementary Information

Magnesium Transporter MgtA revealed as a Dimeric P-type ATPase

Rilee Zeinert[1], Fei Zhou[2], Pedro Franco[3], Jonathan Zöller[3], Henry J. Lessen[4], L. Aravind[5], Julian D. Langer[3], Alexander J. Sodt[4,*], Gisela Storz[1,*], Doreen Matthies[2,*]

[1]Division of Molecular and Cellular Biology, *Eunice Kennedy Shriver* National Institute of Child Health and Human Development, National Institutes of Health, Bethesda MD 20892, USA

[2]Unit on Structural Biology, *Eunice Kennedy Shriver* National Institute of Child Health and Human Development, National Institutes of Health, Bethesda MD 20892, USA

[3]Max Planck Institute of Biophysics, 60438 Frankfurt am Main, Germany

[4]Unit on Membrane Chemical Physics, *Eunice Kennedy Shriver* National Institute of Child Health and Human Development, National Institutes of Health, Bethesda MD 20892, USA

[5]National Center for Biotechnology Information, National Institutes of Health, Bethesda MD 20892, USA

[*]Address correspondence to A.J.S. alexander.sodt@nih.gov, G.S. storzg@mail.nih.gov or D.M. doreen.matthies@nih.gov

bioRxiv preprint doi: https://doi.org/10.1101/2024.02.28.582502; this version posted March 1, 2024. The copyright holder for this preprint (which was not certified by peer review) is the author/funder. This article is a US Government work. It is not subject to copyright under 17 USC 105 and is also made available for use under a CC0 license.

Extended Data Fig. 1	Multisequence alignment of MgtA and MgtB illustrates conserved structural features.
Extended Data Fig. 2	Multisequence alignment of all P-type ATPases.
Extended Data Fig. 3	Amino acids related to catalysis and structural architecture are highly conserved across the P-type ATPase family.
Extended Data Fig. 4	EcMgtA is predicted to be structurally similar to SeMgtA/B and the closest mammalian homologs ATP2C1 and SERCA.
Extended Data Fig. 5	Weaker detergents preserve MgtA native protein-protein interactions.
Extended Data Fig. 6	MgtA forms two distinguishable MW species when purified from *E. coli*.
Extended Data Fig. 7	Negative staining EM analysis of purified MgtA.
Extended Data Fig. 8	Schematic showing cryo-EM data processing workflow for dimeric and monomeric MgtA.
Extended Data Fig. 9	Local and average resolution estimation of the dimeric and monomeric MgtA cryo-EM maps and B-factor distribution of models.
Extended Data Fig. 10	Example regions documenting quality of cryo-EM dimer map for key structural features.
Extended Data Fig. 11	Experimental support of transmembrane borders and lipid distribution.
Extended Data Fig. 12	Structural model is supported by crosslinking and mass spectrometry.
Extended Data Fig. 13	Monomeric MgtA structure adopts a more open conformation relative to the dimer with major changes in TM 1-4, N and A.
Extended Data Fig. 14	The extended N-terminus forms multi-domain electrostatic interactions between the A, P and TM domains.
Extended Data Fig. 15	Mutations at the dimer interface impair Mg^{2+} transport.
Extended Data Fig. 16	Cryo-EM of dimeric MgtA bound to nucleotides reveals extra density in the nucleotide binding pocket.
Extended Data Fig. 17	Local resolution and average resolution of the dimeric MgtA with nucleotides and B-factor distribution of models.
Extended Data Fig. 18	The nucleotide binding pocket of MgtA and other P-type ATPases are structurally similar.
Extended Data Fig. 19	Time sequence of MD simulations of the MgtA dimer with ATP.
Extended Data Fig. 20	HDX-MS analysis of MgtA reveals structural changes upon ATP and ATPγS binding.
Extended Data Fig. 21	Residues D373 and E215 are required for Mg^{2+} transport.
Extended Data Fig. 22	Conservation of key Mg^{2+} binding residues.
Extended Data Fig. 23	Functional analysis of key Mg^{2+} binding residues.
Extended Data Fig. 24	Water accessibility in the TM domain of MgtA.
Extended Data Fig. 25	Predicted and documented interactions of small proteins with P-type ATPase proteins.

Extended Data Table 1	Residues involved in the dimer interface.
Extended Data Table 2	Summary of all mutants generated and their outcome.
Extended Data Table 3	Cryo-EM data collection parameters and analysis.
Extended Data Table 4	Cryo-EM map and model analysis.

Supplementary Data 1	Extended multisequence alignment of all P-type ATPases.
Supplementary Data 2	Primers, plasmids, and strains used in this study.

bioRxiv preprint doi: https://doi.org/10.1101/2024.02.28.582502; this version posted March 1, 2024. The copyright holder for this preprint (which was not certified by peer review) is the author/funder. This article is a US Government work. It is not subject to copyright under 17 USC 105 and is also made available for use under a CC0 license.

Extended Data Movie 1 360 degree view of the dimeric cryo-EM map of *E. coli* Mg^{2+} transporter MgtA.
Extended Data Movie 2 360 degree view of the monomeric cryo-EM map of *E. coli* Mg^{2+} transporter MgtA.
Extended Data Movie 3 Morph between the structural model of a single subunit of the dimeric and monomeric *E. coli* Mg^{2+} transporter MgtA.
Extended Data Movie 4 MD simulation movie of the N-terminal tail of the *E. coli* Mg^{2+} transporter MgtA.
Extended Data Movie 5 MD simulation movie of the *E. coli* Mg^{2+} transporter MgtA showing the full dimer.
Extended Data Movie 6 Zoomed in MD simulation movie of the *E. coli* Mg^{2+} transporter MgtA dimer interface.
Extended Data Movie 7 The *E. coli* Mg^{2+} transporter MgtA dimer with ATP bound.
Extended Data Movie 8 MD simulation movie of the Mg^{2+} ion in the middle of the transmembrane domains of the *E. coli* Mg^{2+} transporter MgtA.

bioRxiv preprint doi: https://doi.org/10.1101/2024.02.28.582502; this version posted March 1, 2024. The copyright holder for this preprint (which was not certified by peer review) is the author/funder. This article is a US Government work. It is not subject to copyright under 17 USC 105 and is also made available for use under a CC0 license.

Extended Data Figures

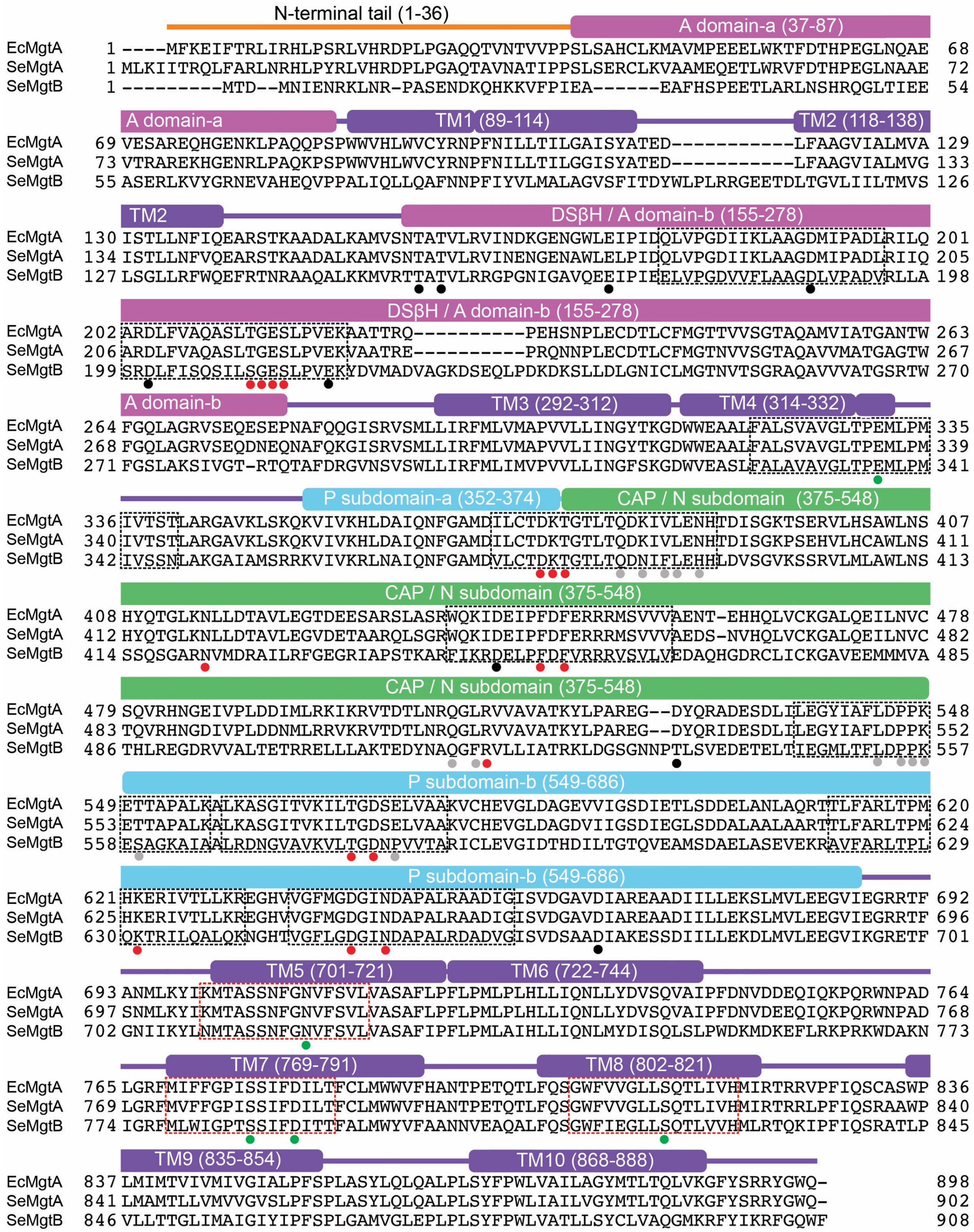

bioRxiv preprint doi: https://doi.org/10.1101/2024.02.28.582502; this version posted March 1, 2024. The copyright holder for this preprint (which was not certified by peer review) is the author/funder. This article is a US Government work. It is not subject to copyright under 17 USC 105 and is also made available for use under a CC0 license.

Extended Data Fig 1 Multisequence alignment of MgtA and MgtB illustrates conserved structural features. Sequence alignment of *E. coli* MgtA (EcMgtA), *S. enterica* serovar Typhimurium MgtA (SeMgtA), and *S. enterica* serovar Typhimurium MgtB (SeMgtB) generated using Clustal Omega. Domains are colored and named according to Fig. 1. Both the canonical P-type ATPase nomenclature and more detailed description of the fold of the domain with boundaries are given. The soluble A domain is split into two regions a and b. The b segment of the A domain is comprised of a Double Stranded beta-Helix fold (DSβH). The soluble P subdomain is a noncontiguous segment comprised of two regions a and b that house the key catalytic residues required for phosphorylation. The N or CAP subdomain is a contiguous sequence that binds the nucleotide and aids in catalysis. The P and N subdomains comprise the Haloacid dehalogenase (HAD) domain. The TM-spanning alpha-helical regions, as determined by a residue's alpha-carbon position residing, on average, within the hydrocarbon bilayer interior of the molecular dynamics simulations (+/- 15 Angstroms from the bilayer midplane) are denoted by TM1-10. Gray circles denote residues present at the dimer interface, red circles denote residues near ATP, green circles denote residues coordinating Mg^{2+} in the transmembrane domain, and black circles denote residues coordinating Mg^{2+} in the cytoplasmic domain in our dimeric cryo-EM structures, as annotated in Fig. 2, 3 and 4. Black dashed boxes indicate sequences conserved across all P-type ATPases as shown in the logos in Extended Data Fig. 3. Red dashed boxes indicate sequences specific to Mg^{2+} importers as shown in the logos in Extended Data Fig. 22.

bioRxiv preprint doi: https://doi.org/10.1101/2024.02.28.582502; this version posted March 1, 2024. The copyright holder for this preprint (which was not certified by peer review) is the author/funder. This article is a US Government work. It is not subject to copyright under 17 USC 105 and is also made available for use under a CC0 license.

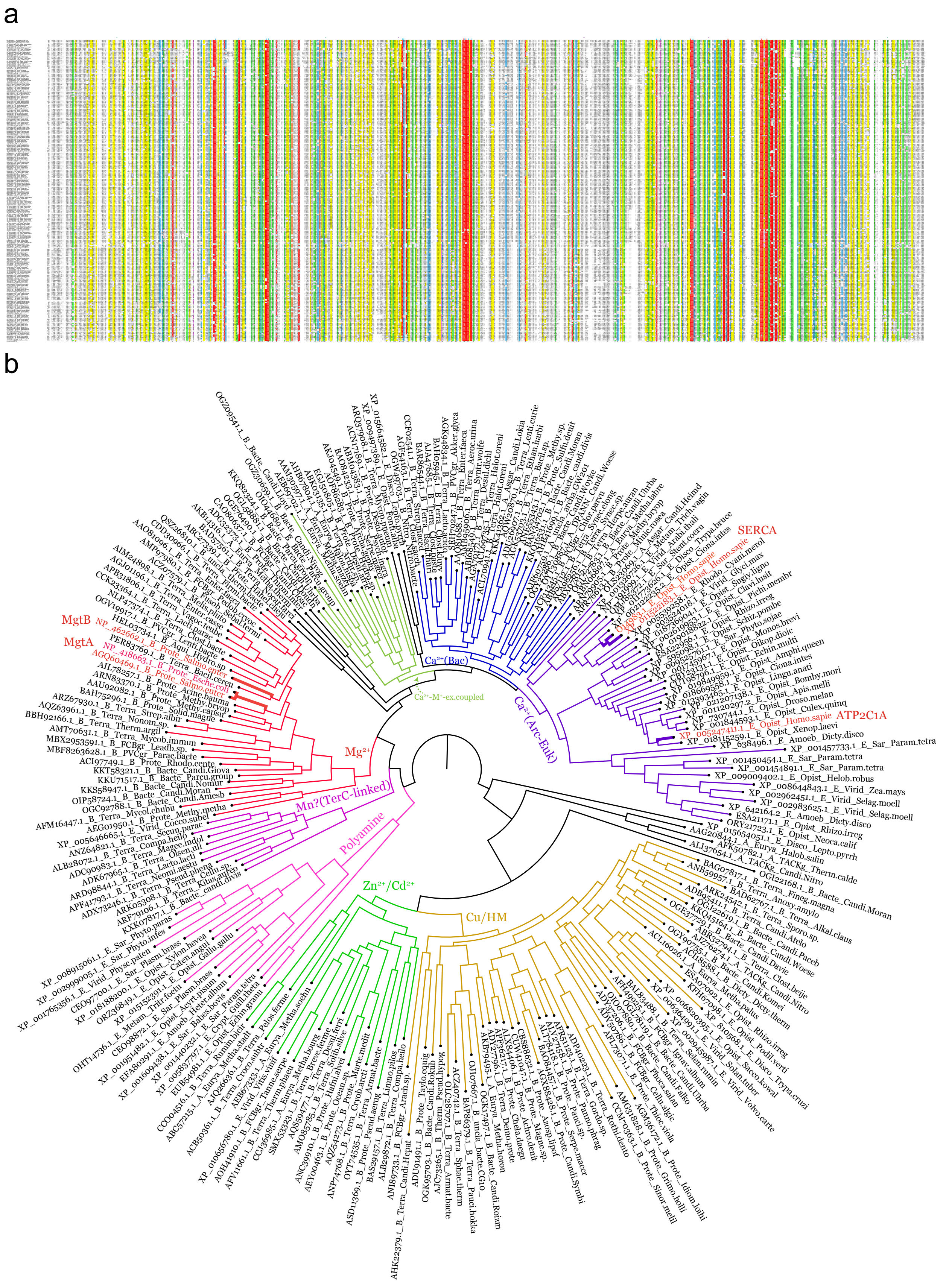

bioRxiv preprint doi: https://doi.org/10.1101/2024.02.28.582502; this version posted March 1, 2024. The copyright holder for this preprint (which was not certified by peer review) is the author/funder. This article is a US Government work. It is not subject to copyright under 17 USC 105 and is also made available for use under a CC0 license.

Extended Data Fig 2 Multisequence alignment of all P-type ATPases. a, A multiple sequence alignment of representatives of the eight major clades of P-type ATPases that are shown in different colors in the tree in **b** (see Supplementary Data 1 for an extended sequence alignment). The first six sequences are *E. coli* MgtA, *S. enterica* MgtA, *S. enterica* MgtB; *H. sapiens* ATP2C1 mutated in Hailey-Hailey disease (the HHD mutations are marked with H above the alignment), *H. sapiens* SERCA1 mutated in Brody's myopathy (the Brody's myopathy mutations are marked with B above the alignment) and *H. sapiens* SERCA3. The alignment is colored according to the 85% consensus shown in the final line and the sidechain type of the consensus position: l, aliphatic; a, aromatic; h, hydrophobic; +, positive; -, negative; c, charged; p, polar; t, tiny; s, small; b, big. The sequences are labeled by their NCBI Genbank accession, followed by taxonomic marker where B is bacteria, A is Archaea, and E is Eukaryota. This is followed by abbreviations of higher-order taxonomic lineage and species. The positions of the catalytic residues are indicated by red dots, while other conserved positions are shown with blue dots. **b,** In the tree, the clades are labeled as per their known or predicted transport substrates. All the distinctly colored clades are supported with IQtree bootstrap sport of 90% or higher.

bioRxiv preprint doi: https://doi.org/10.1101/2024.02.28.582502; this version posted March 1, 2024. The copyright holder for this preprint (which was not certified by peer review) is the author/funder. This article is a US Government work. It is not subject to copyright under 17 USC 105 and is also made available for use under a CC0 license.

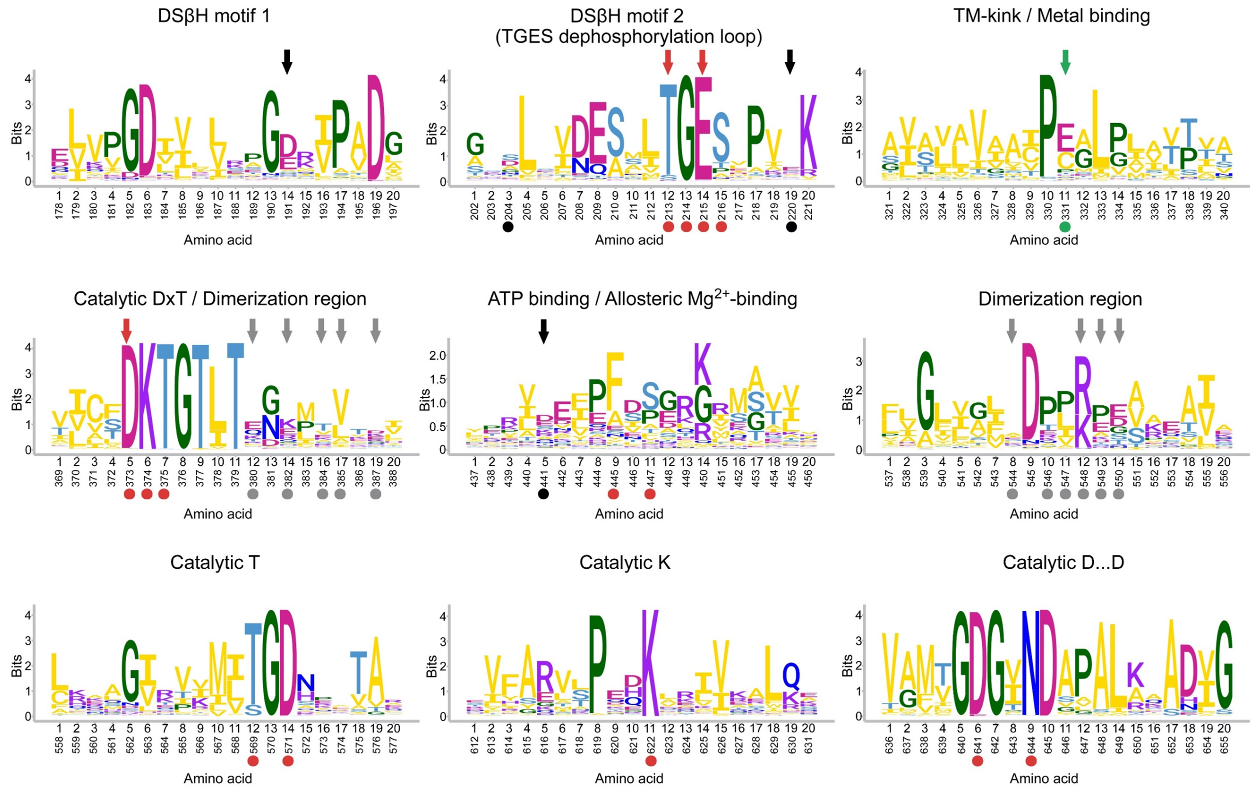

Extended Data Fig 3 Amino acids related to catalysis and structural architecture are highly conserved across the P-type ATPase family. Sequence logos showing conservation of amino acid residues involved in ATP hydrolysis and structural architecture conserved among entire family of P-type ATPases (indicated by black dashed boxes in Extended Data Fig. 1). Letters represent amino acid abbreviations and height represents the probability of conservation in the P-type ATPase family. As in Extended Data Fig. 1, gray circles denote residues present at the dimer interface, red circles denote residues involved in ATP hydrolysis, green circles denote residues coordinating Mg^{2+} in the transmembrane domain, and black circles denote residues coordinating Mg^{2+} in the cytoplasmic domain in our dimeric structures, as annotated in Fig. 2, 3 and 4. Gray, red, green and black arrows, respectively, indicate residues located at the dimer interface, involved in ATP hydrolysis, coordinating the transmembrane or cytoplasmic Mg^{2+}, which were mutated in subsequent experiments.

bioRxiv preprint doi: https://doi.org/10.1101/2024.02.28.582502; this version posted March 1, 2024. The copyright holder for this preprint (which was not certified by peer review) is the author/funder. This article is a US Government work. It is not subject to copyright under 17 USC 105 and is also made available for use under a CC0 license.

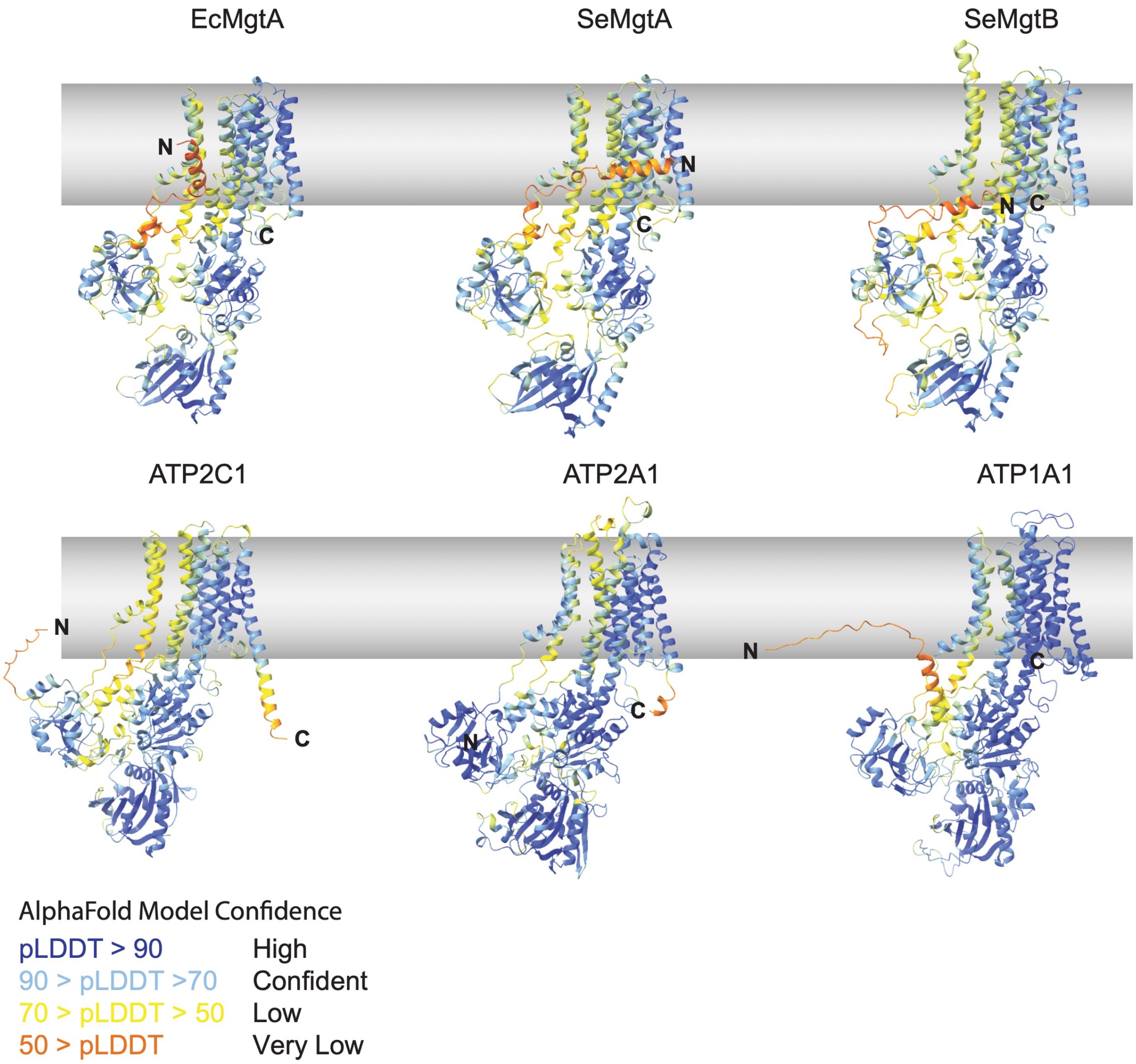

Extended Data Fig 4 EcMgtA is predicted to be structurally similar to SeMgtA/B and the closest mammalian homologs ATP2C1 and SERCA. AlphaFold2 model for MgtA and related transporters predict a similar overall structural architecture. Models are displayed based on AlphaFold2 predictions for *E. coli* MgtA (EcMgtA), *S. enterica* MgtA (SeMgtA) and *S. enterica* MgtB (SeMgtB). The highest confidence *Homo sapiens* ATP2C1 (a Ca^{2+}/Mn^{2+} transporter) and SERCA (ATP2A1) models are shown along with the highest confidence model for *H. sapiens* Na^{+}/K^{+} transporter (ATP1A1), which also has an N-terminal tail. The per-residue confidence metric (pLDDT) was used to color the models. Regions expected to be modelled with higher confidence are blue while regions with lower confidence are orange as indicated in the legend. The structures of all N-termini are of low confidence.

bioRxiv preprint doi: https://doi.org/10.1101/2024.02.28.582502; this version posted March 1, 2024. The copyright holder for this preprint (which was not certified by peer review) is the author/funder. This article is a US Government work. It is not subject to copyright under 17 USC 105 and is also made available for use under a CC0 license.

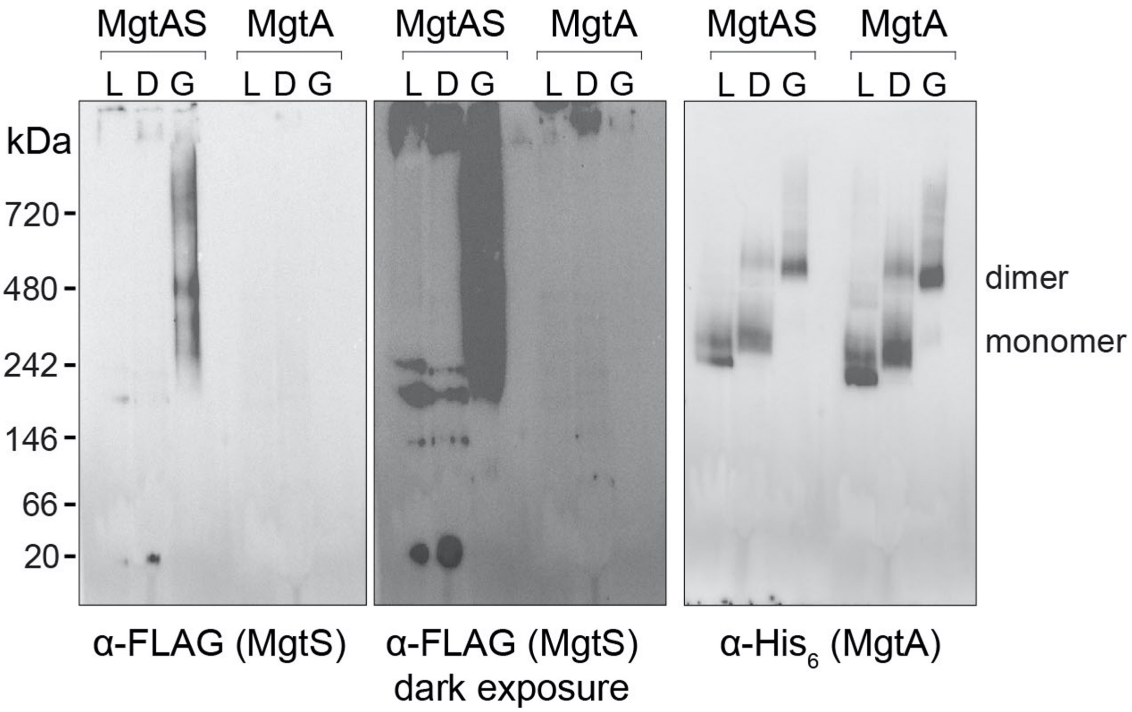

Extended Data Fig 5 Weaker detergents preserve MgtA native protein-protein interactions. Solubilization of membranes expressing MgtA or MgtAS with detergents of varying strengths show differences in native protein interactions when analyzed by G250 Blue-Native gel and Western blot analysis. Membranes from cells overexpressing MgtA or MgtAS were solubilized with the detergents LMNG (L), DDM (D), or GDN (G) prior to Blue-Native PAGE and Western blot analysis using α-FLAG antibodies against tagged MgtS (left two panels; middle panel is a longer exposure of the left panel) or α-His_6 antibodies against tagged MgtA (right panel).

bioRxiv preprint doi: https://doi.org/10.1101/2024.02.28.582502; this version posted March 1, 2024. The copyright holder for this preprint (which was not certified by peer review) is the author/funder. This article is a US Government work. It is not subject to copyright under 17 USC 105 and is also made available for use under a CC0 license.

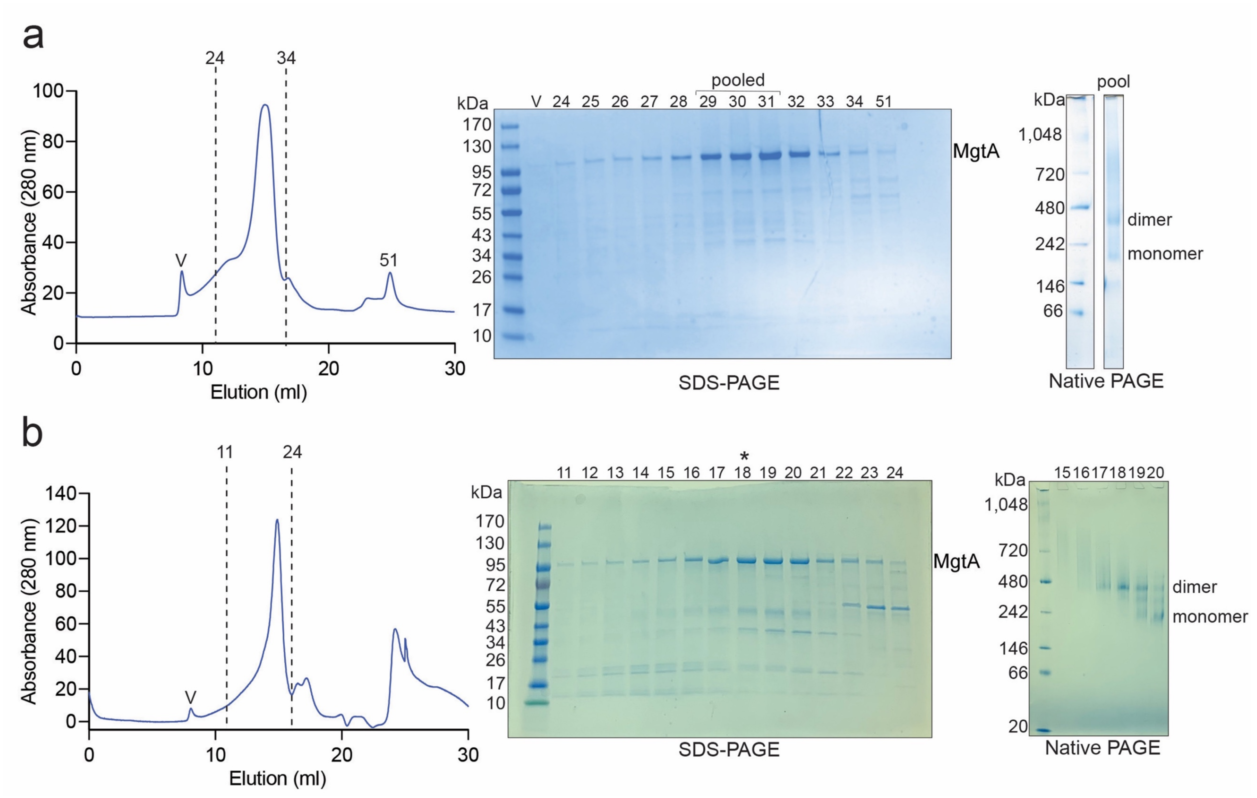

Extended Data Fig 6 MgtA forms two distinguishable MW species when purified from *E. coli*. a, Purification of MgtA results in two distinct MW protein complexes. SEC profile (left) of MgtA used to solve the dimer and monomer structures of MgtA in Fig. 1. Fractions were analyzed by SDS-PAGE (middle) and Blue-Native PAGE gels (right). V indicates the void volume of the SEC column. Fractions that were used to solve the structure are referred to as pooled. **b**, Dimeric MgtA can be separated from monomeric MgtA. SEC profile (left) of MgtA used to solve the nucleotide bound dimer structures of MgtA in Fig. 3 and Extended Data Fig. 16. Fractions were analyzed by SDS-PAGE (middle) and Blue-Native PAGE gels (right). Fraction 18 which possessed predominantly dimer species was used for structural analysis.

bioRxiv preprint doi: https://doi.org/10.1101/2024.02.28.582502; this version posted March 1, 2024. The copyright holder for this preprint (which was not certified by peer review) is the author/funder. This article is a US Government work. It is not subject to copyright under 17 USC 105 and is also made available for use under a CC0 license.

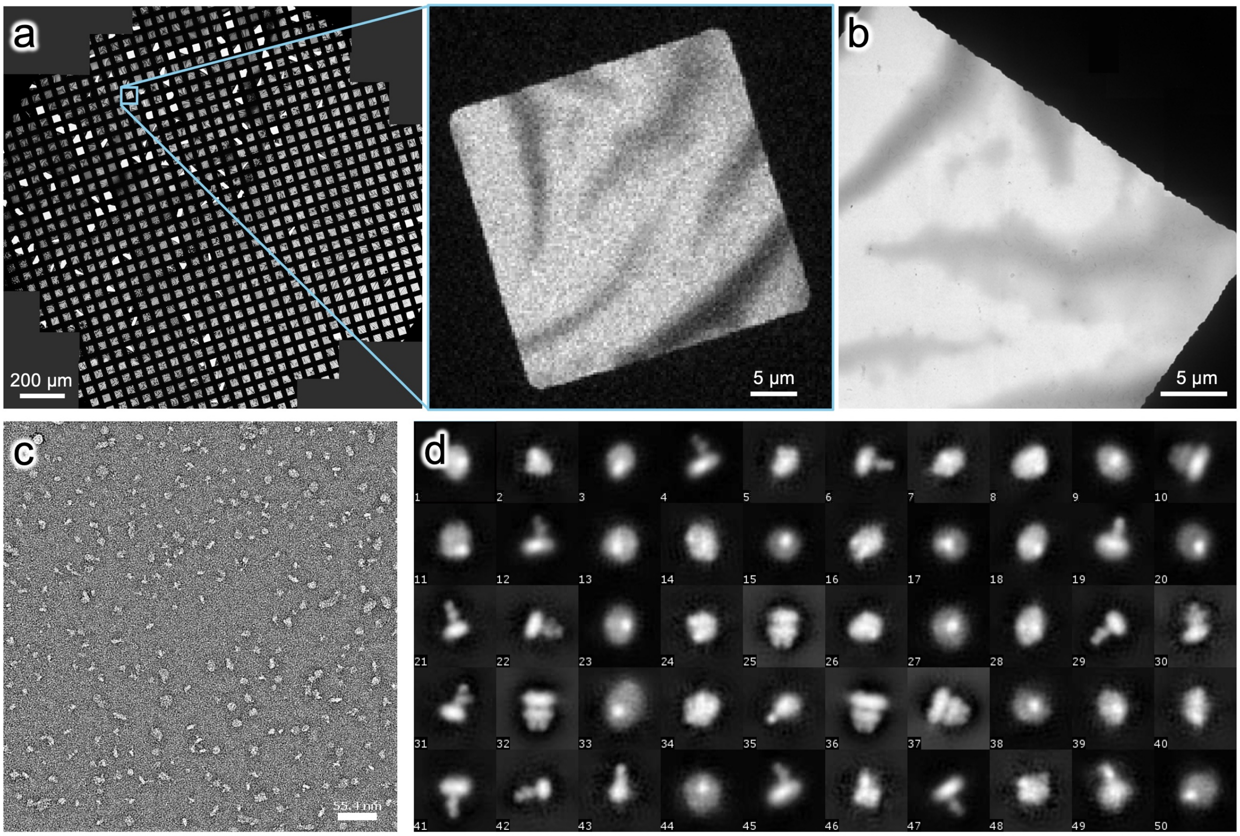

Extended Data Fig 7 Negative staining EM analysis of purified MgtA. a, Low magnification atlas of a negative staining grid of MgtA diluted to 0.01 mg/ml of purified MgtA and zoom in into a suitable grid square for data collection with a decent thickness of staining in the lighter regions. **b**, Medium magnification image of part of the grid square highlighted in a. **c**, Representative micrograph of negatively stained single particles shows no protein aggregation and sufficient particle density. **d**, 2D class averages with a box size of 84 px (~255 Å) indicating larger particles as well as monomeric looking particles in different orientations.

bioRxiv preprint doi: https://doi.org/10.1101/2024.02.28.582502; this version posted March 1, 2024. The copyright holder for this preprint (which was not certified by peer review) is the author/funder. This article is a US Government work. It is not subject to copyright under 17 USC 105 and is also made available for use under a CC0 license.

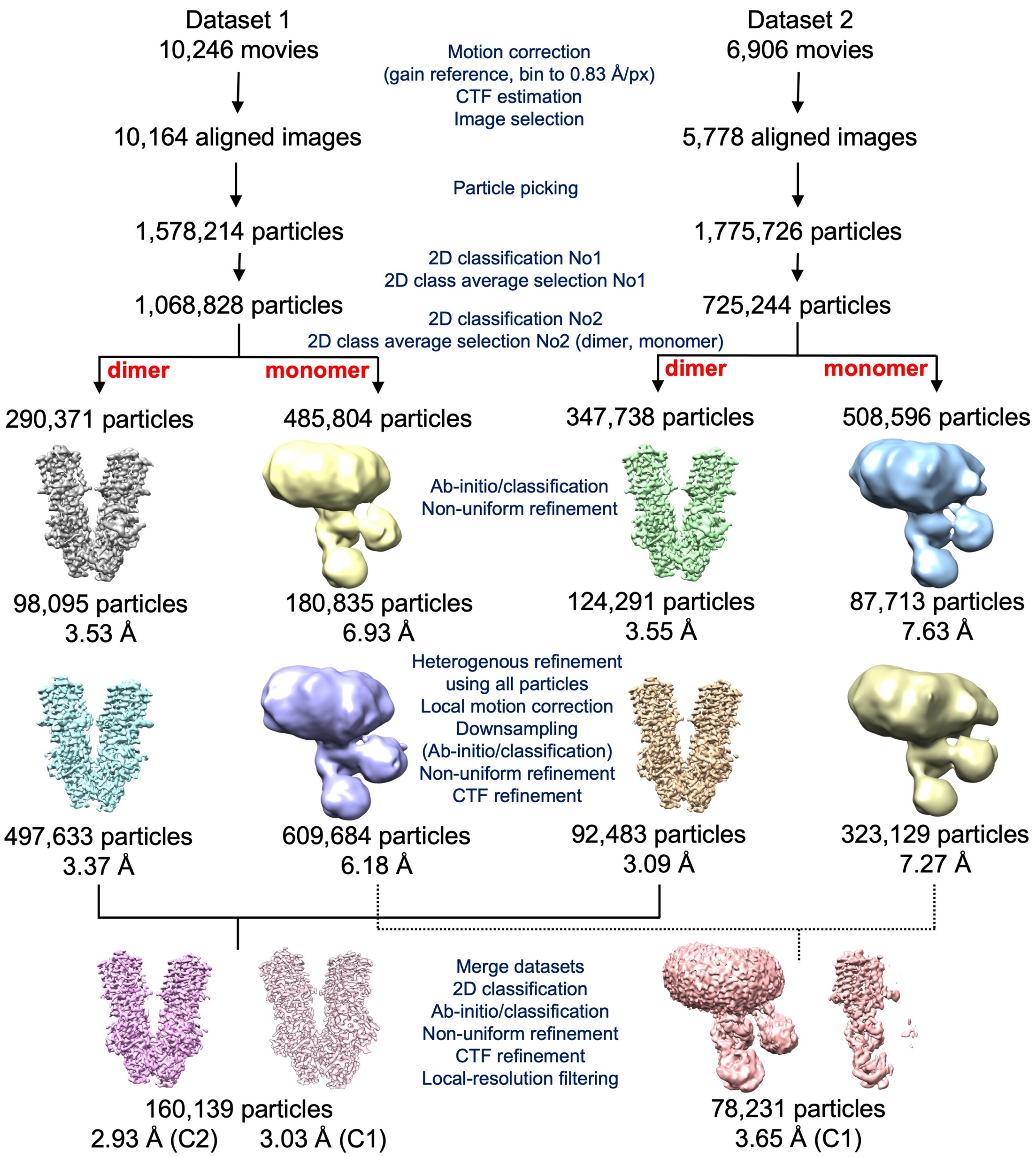

Extended Data Fig 8 Schematic showing cryo-EM data processing workflow for dimeric and monomeric MgtA. Two datasets for EcMgtA in the presence of 5 mM $MgCl_2$ were initially processed separately before merging dimeric and monomeric particles from each dataset followed by additional classification and refinement steps to obtain the final reconstructions used for model building. Number of movies and particles as well as the by cryoSPARC estimated resolution is indicated (also see Extended Data Fig. 9 and Extended Data Tables 3 and 4).

bioRxiv preprint doi: https://doi.org/10.1101/2024.02.28.582502; this version posted March 1, 2024. The copyright holder for this preprint (which was not certified by peer review) is the author/funder. This article is a US Government work. It is not subject to copyright under 17 USC 105 and is also made available for use under a CC0 license.

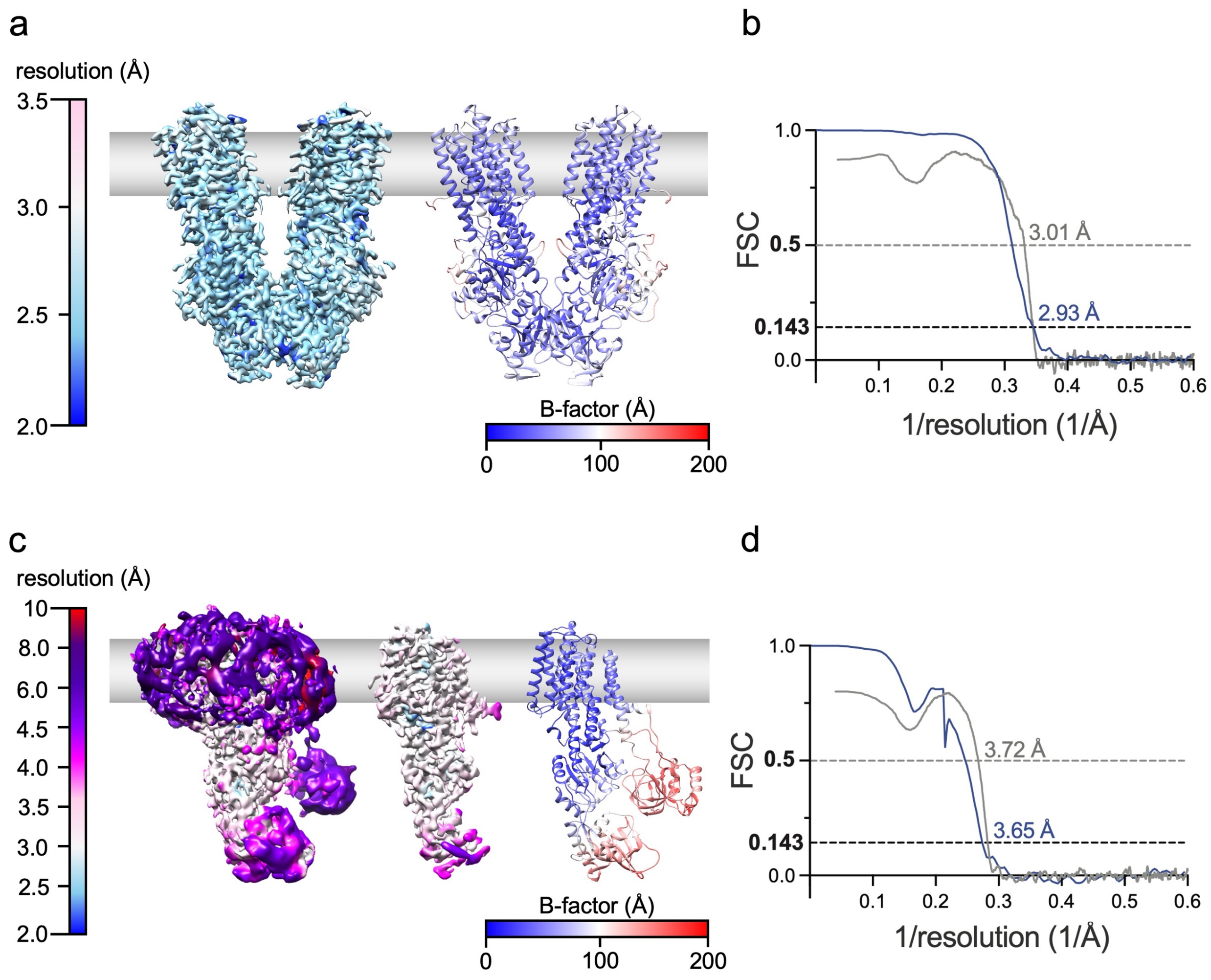

Extended Data Fig 9 Local and average resolution estimation of the dimeric and monomeric MgtA cryo-EM maps and B-factor distribution of models. a, Final dimer reconstruction filtered and colored to local resolution (left) and fitted model colored according to B-factor distribution (right) indicating rigid and more flexible regions of the complex. **b**, Fourier Shell Correlation (FSC) curve of the final dimer reconstruction of MgtA in blue indicating an average resolution of 2.93 Å according to the FSC=0.143 criterion. FSC between the final dimer map and fitted model is shown in gray. **c**, Final monomer reconstruction filtered and colored to local resolution at different thresholds (left and middle) and fitted model colored according to B-factor distribution (right) indicating rigid and more flexible regions. **d**, FSC curve of the final monomer reconstruction of MgtA in blue indicating an average resolution of 3.65 Å according to the FSC=0.143 criterion. FSC between the final monomer map and fitted model is shown in gray.

bioRxiv preprint doi: https://doi.org/10.1101/2024.02.28.582502; this version posted March 1, 2024. The copyright holder for this preprint (which was not certified by peer review) is the author/funder. This article is a US Government work. It is not subject to copyright under 17 USC 105 and is also made available for use under a CC0 license.

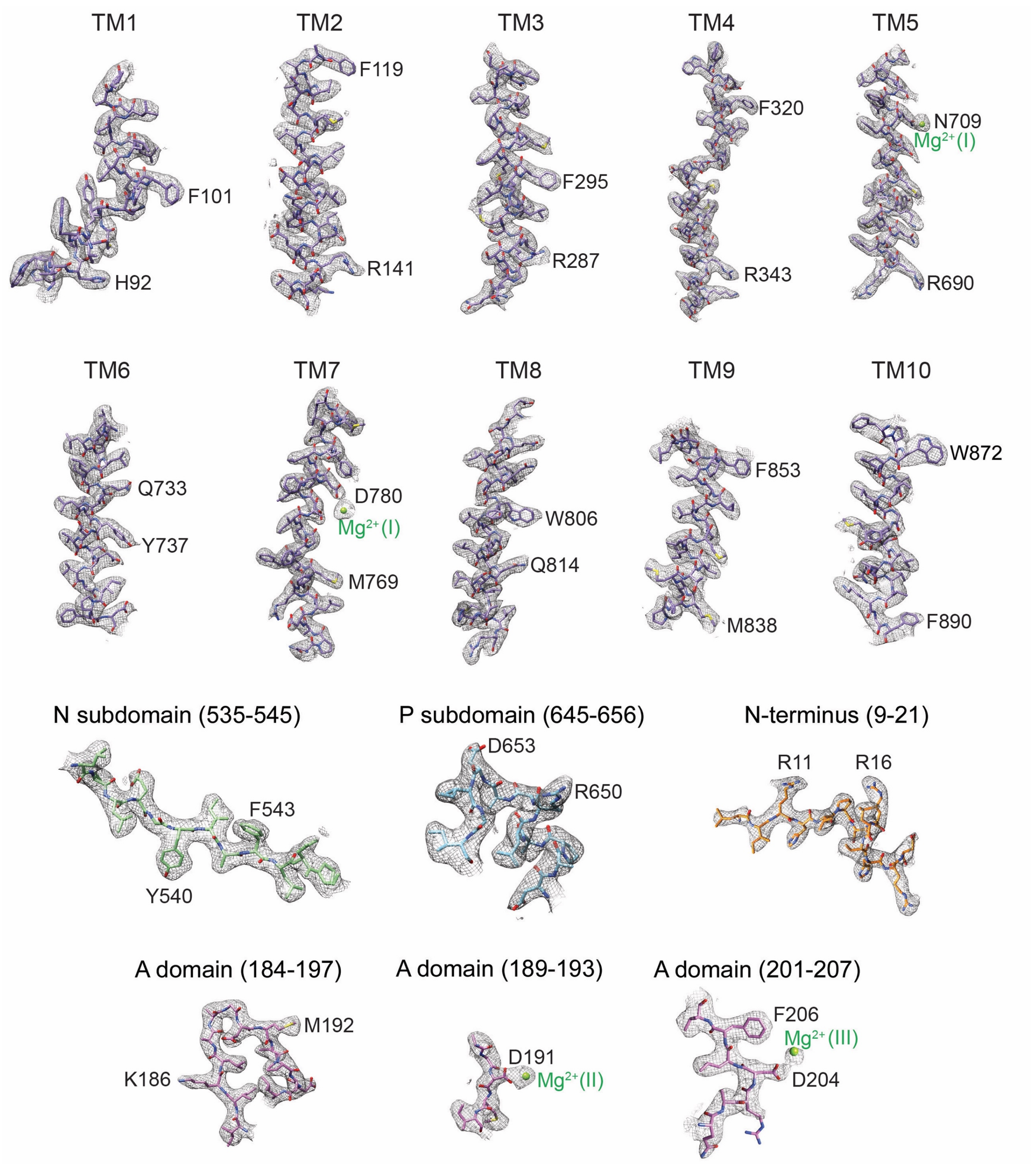

Extended Data Fig 10 Example regions documenting quality of cryo-EM dimer map for key structural features. Cryo-EM density and atomic model of the TM segments (1-10), soluble domains (A, P, N), N-terminus, and Mg^{2+} ions colored as in Fig. 1. Atomic model of each structural element is shown in stick representation, the atoms are colored by heteroatom within the cryo-EM density and the corresponding density is represented in gray mesh.

bioRxiv preprint doi: https://doi.org/10.1101/2024.02.28.582502; this version posted March 1, 2024. The copyright holder for this preprint (which was not certified by peer review) is the author/funder. This article is a US Government work. It is not subject to copyright under 17 USC 105 and is also made available for use under a CC0 license.

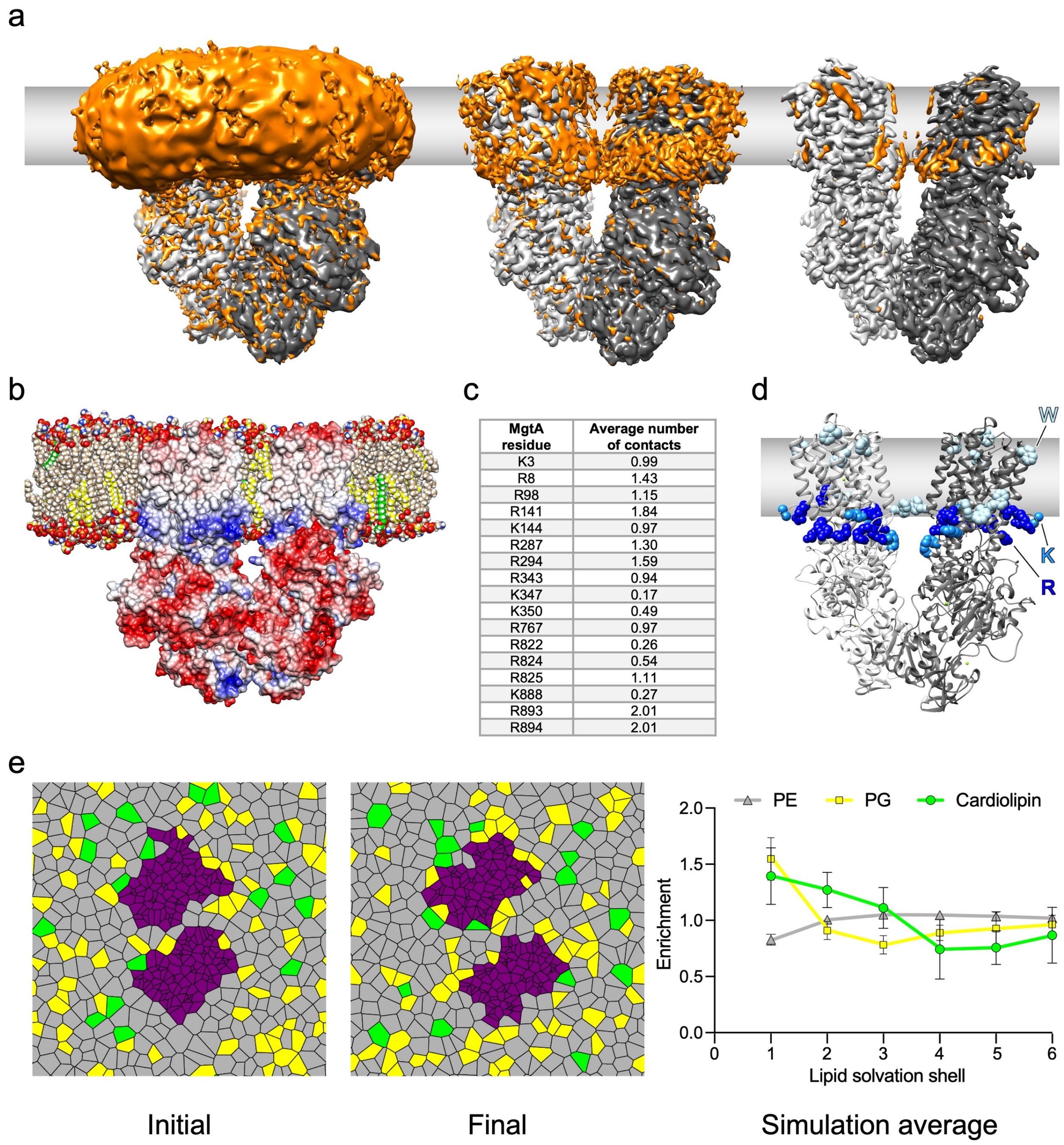

MgtA residue	Average number of contacts
K3	0.99
R8	1.43
R98	1.15
R141	1.84
K144	0.97
R287	1.30
R294	1.59
R343	0.94
K347	0.17
K350	0.49
R767	0.97
R822	0.26
R824	0.54
R825	1.11
K888	0.27
R893	2.01
R894	2.01

Extended Data Fig 11 Experimental support of transmembrane borders and lipid distribution. a, Side views of cryo-EM maps at different thresholds showing extra densities in orange corresponding to the detergent micelle, detergent molecules, or potential co-purified lipids near the transmembrane region. **b**, Side view of MgtA simulated in a native lipid environment displayed in surface representation colored by electrostatic potential (UCSF Chimera coloring varies from red [-10 kcal/mol/e] to blue [+10 kcal/mol/e] with distance-dependent dielectric constant 4, distance from surface 1.4). Phospholipids corresponding to phosphatidylethanolamine (PE), phosphatidylglycerol (PG) and cardiolipin are colored

bioRxiv preprint doi: https://doi.org/10.1101/2024.02.28.582502; this version posted March 1, 2024. The copyright holder for this preprint (which was not certified by peer review) is the author/funder. This article is a US Government work. It is not subject to copyright under 17 USC 105 and is also made available for use under a CC0 license.

tan, yellow, and green, respectively. **c**, Arginine and lysine residues that interact with anionic lipids during at least 20% of the simulation, with cutoff enclosing the first peak of the radial distribution function. **d**, Side view of MgtA with arginine (R), lysine (K), and tryptophan (W) residues near the lipid membrane borders highlighted in blue spheres. **e**, Voronoi decomposition of lipid centers-of-geometry in the cytoplasmic leaflet initially (initial) and at the end of the simulation (final). Yellow and green are anionic PG and cardiolipin lipids, respectively. At right, average enrichment or depletion of lipids based on solvation shell as assigned by Voronoi decomposition averaged over the trajectory.

bioRxiv preprint doi: https://doi.org/10.1101/2024.02.28.582502; this version posted March 1, 2024. The copyright holder for this preprint (which was not certified by peer review) is the author/funder. This article is a US Government work. It is not subject to copyright under 17 USC 105 and is also made available for use under a CC0 license.

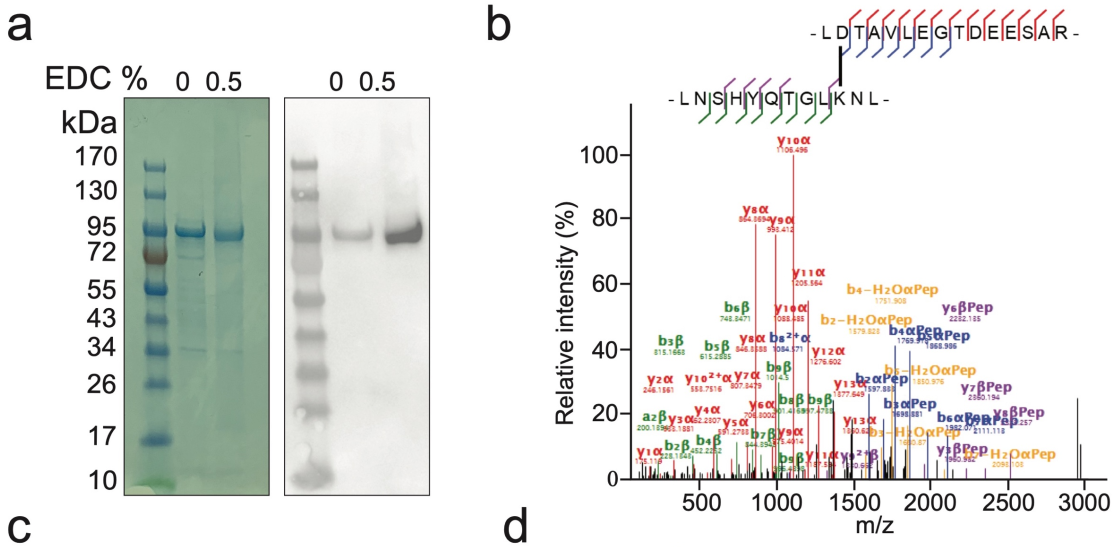

c

Crosslinked residues	Distance (Å)
K352-E675	3.5
K414-D418	4.8
K374-D381	8.7
K55-E52	8.4
K55-D58	10.0
K382-E386	10.8
K80-E70	12.5
K518-E538	14.2
K150-E275	17.6
K676-E682	18.3
K80-E68	19.5
K144-E667	23.0
K374-D368	25.3
K144-E275	28.0
K144-E277	33.2
K548-E538	35.2
K166-D526	40.8
K144-E215	45.5

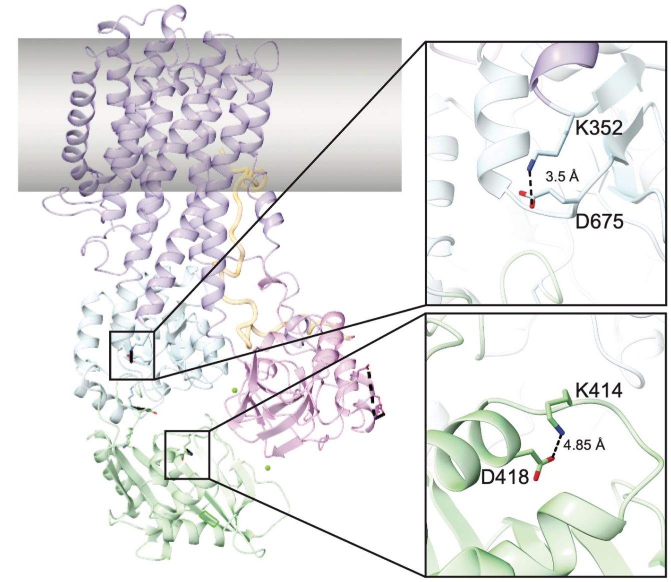

Extended Data Fig 12 Structural model is supported by crosslinking and mass spectrometry. a, MgtAS purified with GDN and crosslinked with EDC. Crosslinked samples were analyzed by SDS-PAGE followed by Coomassie staining (left) or Western blot analysis using α-His_6 antibodies against MgtA (right). **b**, High-quality MS/MS spectrum for a representative crosslinked peptide listed in panel **c**. Matched b- and y- ions are highlighted for the respective peptides, according to their sequences illustrated above. **c**, Panel of high confidence crosslinks identified by mass spectrometry and their distances measured within a monomeric subunit of the dimeric MgtA structure. **d**, Two representative crosslinks of <10 Å mapped onto a single subunit of the atomic model of the MgtA dimer for viewing purposes. Side chains are displayed in stick representation and colored by heteroatom. The color scheme of the model is based on Fig. 1 with transparency applied for visualization of the crosslinks.

bioRxiv preprint doi: https://doi.org/10.1101/2024.02.28.582502; this version posted March 1, 2024. The copyright holder for this preprint (which was not certified by peer review) is the author/funder. This article is a US Government work. It is not subject to copyright under 17 USC 105 and is also made available for use under a CC0 license.

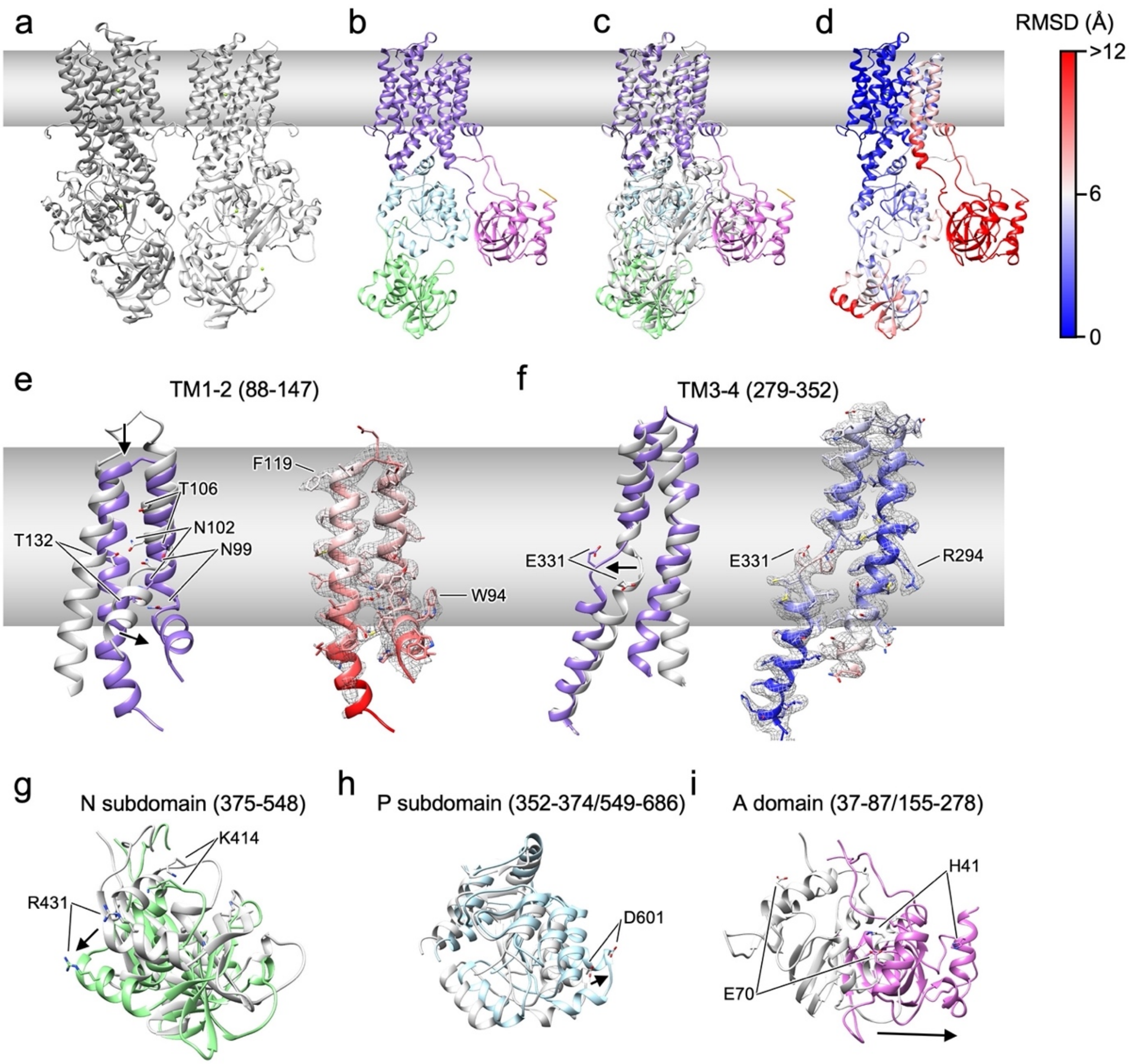

Extended Data Fig 13 Monomeric MgtA structure adopts a more open conformation relative to the dimer with major changes in TM1-4, N and A. a, Dimeric structure with two subunits in two different gray tones and Mg^{2+} ions as green spheres. **b**, Monomeric structure colored as in Fig. 1. **c-d**, To visualize structural changes the colored monomer and a single dimer subunit in gray were superimposed (**c**) and RMSD was calculated. The monomer structure is shown colored by RMSD indicating the largest differences when comparing the two structures by high RMSD values in red. **e-f**, Structural differences between the dimer (gray) and monomer (purple) for TM1-2 (**e**) and TM3-4 (**f**). The monomer colored according to RMSD is on the right with densities in mesh indicating the fit. **g-i**, Structural differences between the soluble domains of the dimer (gray) and monomer (colored).

bioRxiv preprint doi: https://doi.org/10.1101/2024.02.28.582502; this version posted March 1, 2024. The copyright holder for this preprint (which was not certified by peer review) is the author/funder. This article is a US Government work. It is not subject to copyright under 17 USC 105 and is also made available for use under a CC0 license.

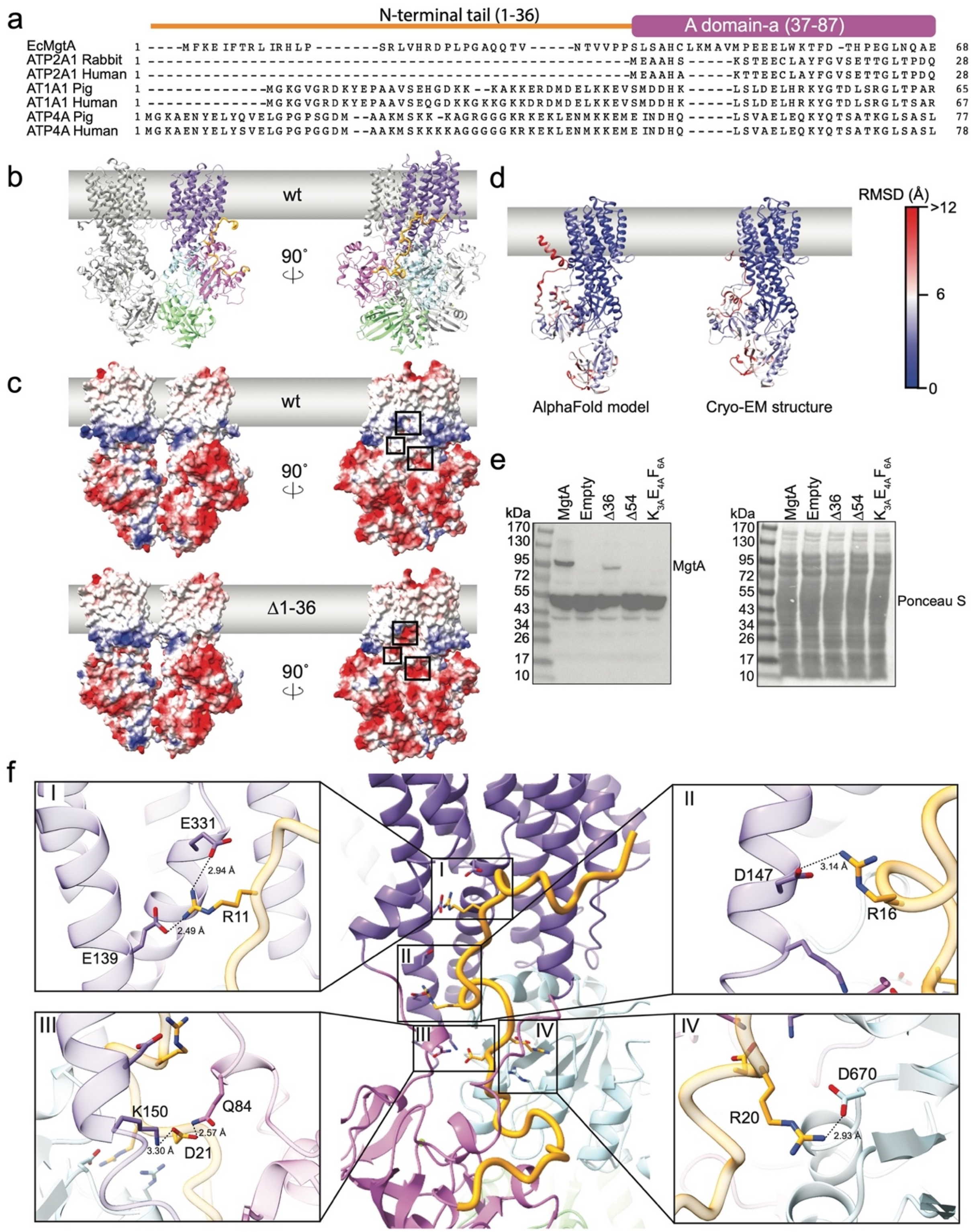

Extended Data Fig 14 The extended N-terminus forms multi-domain electrostatic interactions between the A, P and TM domains. a, Reduced alignment showing an extended N-terminus is present

bioRxiv preprint doi: https://doi.org/10.1101/2024.02.28.582502; this version posted March 1, 2024. The copyright holder for this preprint (which was not certified by peer review) is the author/funder. This article is a US Government work. It is not subject to copyright under 17 USC 105 and is also made available for use under a CC0 license.

in MgtA, Na^+/K^+ ATPases, and H^+/K^+ ATPases, but not the Ca^{2+} pump SERCA. The sequence alignment of *E. coli* MgtA (EcMgtA), ATP2A1 (SERCA) from rabbit and human, AT1A1 (Na^+/K^+) from pig and human, and ATP4A (H^+/K^+) from pig and human was generated using Clustal Omega. Domains are colored and named according to Fig. 1. **b**, Front and side view of the EcMgtA dimer structure colored as in Fig. 1 with the N-terminus highlighted in a thicker loop. The N-terminus of MgtA forms electrostatic interactions with the A, P and TM domains. **c**, Front and side view of the surface representation of the electrostatic potential is displayed for wt and the deletion of the N-terminus (Δ1-36). The structure is rotated 90˚ to highlight the negatively charged patch that is interacting with the N-terminus. **d**, AlphaFold is unable to predict the N-terminus of MgtA. To visualize structural differences between the AlphaFold model and dimeric MgtA cryo-EM structure the RMSD was calculated between a single subunit. Both the AlphaFold model and a single subunit of the dimeric MgtA structure are shown colored by RMSD. **e**, Mutations of the N-terminus reduce levels of MgtA. Cells were grown uninduced (- IPTG) overnight at 37˚C in LB supplemented with 100 mM $MgSO_4$ and normalized in lysis buffer prior to western blot analysis with polyclonal anti-MgtA antibodies. Ponceau S-stained membrane serves as a loading control. **f**, Close-up views of sites I-IV which mark salt bridges formed between the N-terminus and the various domains of MgtA.

bioRxiv preprint doi: https://doi.org/10.1101/2024.02.28.582502; this version posted March 1, 2024. The copyright holder for this preprint (which was not certified by peer review) is the author/funder. This article is a US Government work. It is not subject to copyright under 17 USC 105 and is also made available for use under a CC0 license.

a

Mutant	Residue mutations
5 mer	Q380A V384A L385A L544K E549K
6 mer	Q380A K382A V384A L385A L544K E549K
7 mer	Q380A K382A V384A L385A L544K E549K E582K
11 mer	Q380G K382A V384A L385A N387G Q508A L510A L544S K548A E549A T550A

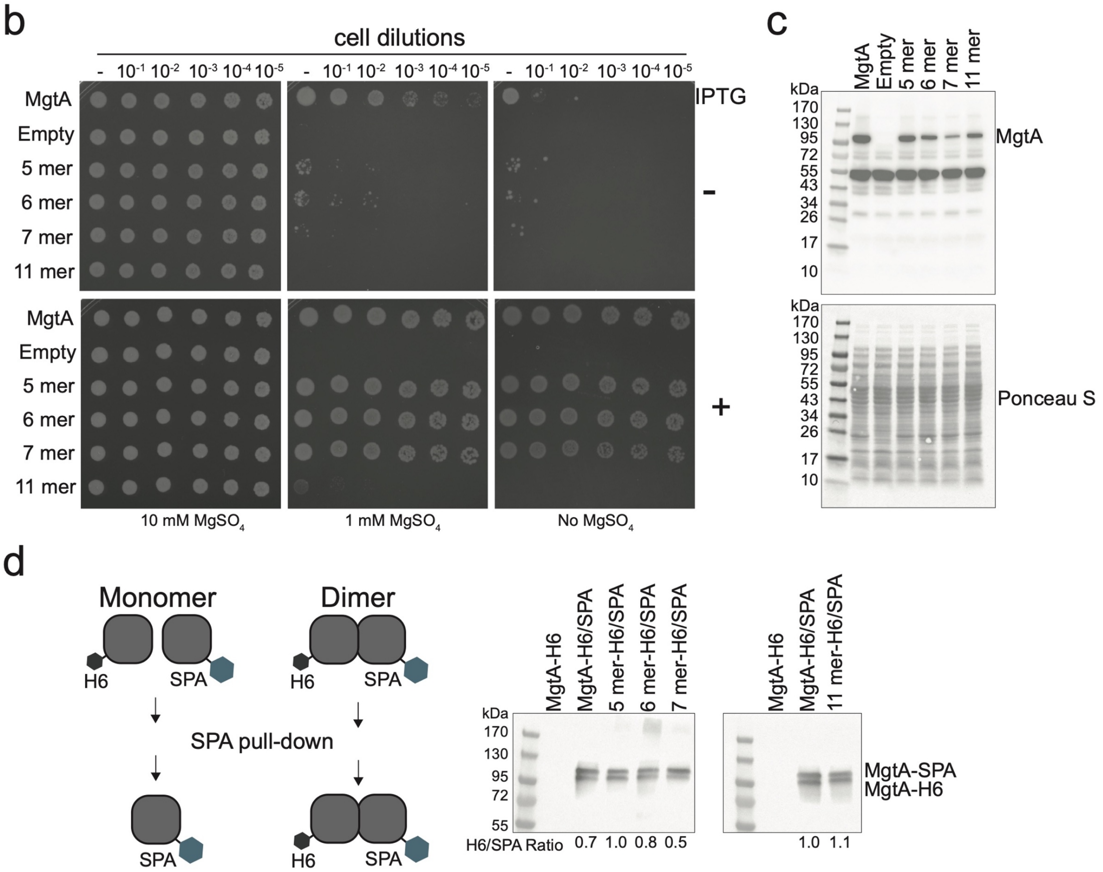

Extended Data Fig 15 Mutations at the dimer interface impair Mg^{2+} transport. a, Summary table of residues mutated at the dimer interface. **b**, Mutations at the dimer interface impair Mg^{2+} ion translocation as visualized by complementation using a Mg^{2+}-auxotrophic *E. coli* strain. Overnight cultures were serial diluted and spotted onto LB agar plates supplemented with the indicated concentrations of $MgSO_4$ with (+) and without (-) 0.1 mM IPTG for induction and grown at 37˚C prior to imaging. **c**, Mutations at the dimer interface reduce levels of MgtA. Cells from the indicated strains were grown uninduced (- IPTG) overnight at 37˚C in LB supplemented with 100 mM $MgSO_4$ and normalized in lysis buffer prior to Western blot analysis with polyclonal anti-MgtA antibodies. Ponceau S-stained membrane serves as a loading control. **d**, The dimer is resistant to directed mutations. Dimer mutants were copurified using differentially tagged MgtA derivatives (as described in Fig. 2b). Proteins were visualized by Western blot analysis using polyclonal anti-MgtA antibodies.

bioRxiv preprint doi: https://doi.org/10.1101/2024.02.28.582502; this version posted March 1, 2024. The copyright holder for this preprint (which was not certified by peer review) is the author/funder. This article is a US Government work. It is not subject to copyright under 17 USC 105 and is also made available for use under a CC0 license.

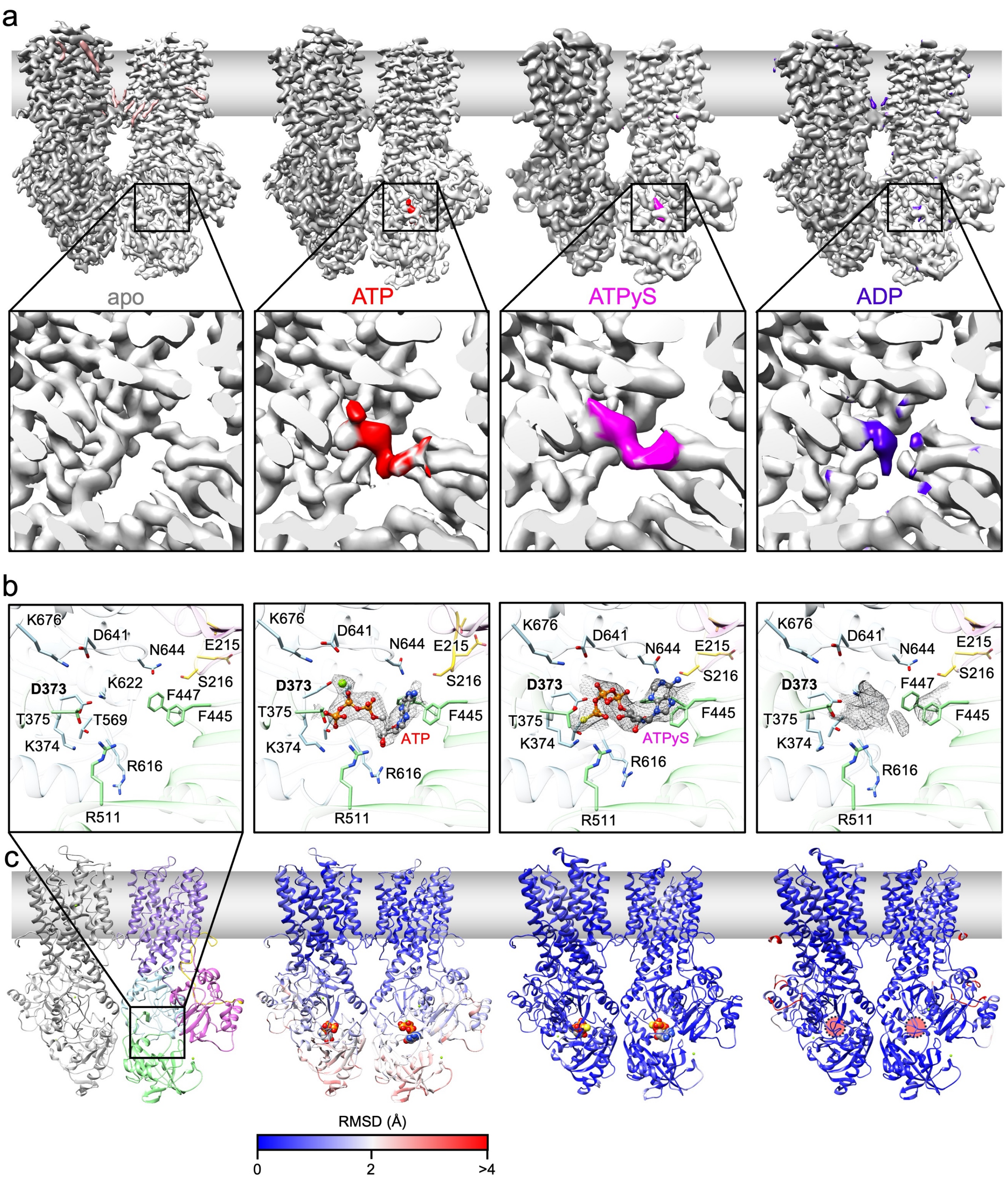

Extended Data Fig 16 Cryo-EM of dimeric MgtA bound to nucleotides reveals extra density in the nucleotide binding pocket. a, Side view of cryo-EM maps of the EcMgtA dimer in the presence of 5 mM $MgCl_2$, and nucleotides. The map of the apo structure is the same as in Fig. 1. Maps are shown in gray and the extra densities are highlighted in color corresponding to the respective nucleotides for MgtA in the presence of 5 mM ATP (red), 5 mM ATPγS (pink), and 5 mM ADP (purple). The average

bioRxiv preprint doi: https://doi.org/10.1101/2024.02.28.582502; this version posted March 1, 2024. The copyright holder for this preprint (which was not certified by peer review) is the author/funder. This article is a US Government work. It is not subject to copyright under 17 USC 105 and is also made available for use under a CC0 license.

resolution of the respective maps are: apo 2.93 Å, ATP 3.72 Å, ATPγS 3.87 Å, and ADP 3.75 Å. **b**, A zoom in into the nucleotide binding site with selected residues shown in stick representation while the nucleotides are in ball-and-stick. Cryo-EM density near the nucleotide is shown in gray mesh. **c**, Dimeric structural models colored according to RMSD when compared to the apo dimeric structure and nucleotides in spheres. For the structure with ATP, residues F447, F445 and N415 from the nucleotide binding subdomain interact with the adenine group of ATP, while F445 appears to form a pi-pi interaction with the adenine and N415 a hydrogen bond with nitrogen N7 of the ATP adenine component. An additional small, isolated density 3 Å from the N6 nitrogen might be a water molecule or ion. Nucleotide binding subdomain residue R511 interacts with the ATP ribose component through a water molecule. Several residues, including D373, K374, and T375 in the phosphorylation subdomain, which form the conserved DxT catalytic motif required for ATP hydrolysis, as well as T569, D571, and K622, all of which interact with the β- and γ-phosphate groups of the ATP molecule. A continuous density to T375 and T569 indicates a close interaction between the γ-phosphate and these two residues. A Mg^{2+} was assigned to a strong round density observed near the β-phosphate. This is near D373, which is known to be the phosphorylated catalytic residue.

bioRxiv preprint doi: https://doi.org/10.1101/2024.02.28.582502; this version posted March 1, 2024. The copyright holder for this preprint (which was not certified by peer review) is the author/funder. This article is a US Government work. It is not subject to copyright under 17 USC 105 and is also made available for use under a CC0 license.

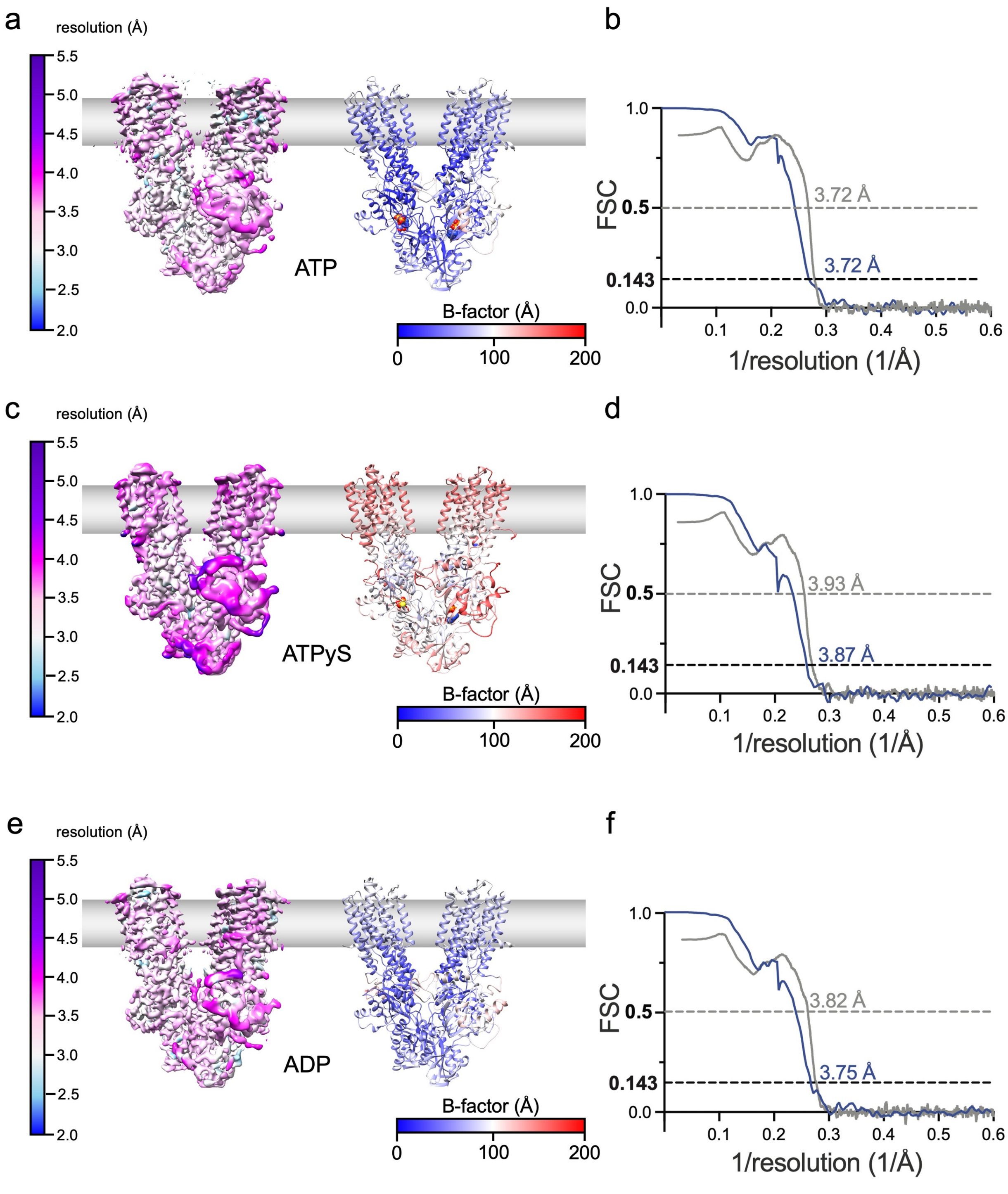

Extended Data Fig 17 Local resolution and average resolution of the dimeric MgtA with nucleotides and B-factor distribution of models. a, Final dimer reconstruction in the presence of 5 mM ATP filtered and colored to local resolution (left) and fitted model colored according to B-factor distribution (right) indicating rigid and more flexible regions of the complex. **b**, Fourier Shell Correlation (FSC) curve of the final dimer reconstruction of MgtA with ATP in blue indicating an average resolution of 3.72 Å

bioRxiv preprint doi: https://doi.org/10.1101/2024.02.28.582502; this version posted March 1, 2024. The copyright holder for this preprint (which was not certified by peer review) is the author/funder. This article is a US Government work. It is not subject to copyright under 17 USC 105 and is also made available for use under a CC0 license.

according to the FSC=0.143 criterion. FSC between the final ATP dimer map and fitted model is shown in gray. **c**, Final dimer reconstruction in the presence of 5 mM ATPγS filtered and colored to local resolution (left) and fitted model colored according to B-factor distribution (right) indicating rigid and more flexible regions of the complex. **d**, FSC curve of the final dimer reconstruction of MgtA with ATPγS in blue indicating an average resolution of 3.87 Å according to the FSC=0.143 criterion. FSC between the final ATPγS dimer map and fitted model is shown in gray. **e**, Final dimer reconstruction in the presence of 5 mM ADP filtered and colored to local resolution (left) and fitted model colored according to B-factor distribution (right) indicating rigid and more flexible regions of the complex. **f**, FSC curve of the final dimer reconstruction of MgtA with ADP in blue indicating an average resolution of 3.75 Å according to the FSC=0.143 criterion. FSC between the final ADP dimer map and fitted model is shown in gray.

bioRxiv preprint doi: https://doi.org/10.1101/2024.02.28.582502; this version posted March 1, 2024. The copyright holder for this preprint (which was not certified by peer review) is the author/funder. This article is a US Government work. It is not subject to copyright under 17 USC 105 and is also made available for use under a CC0 license.

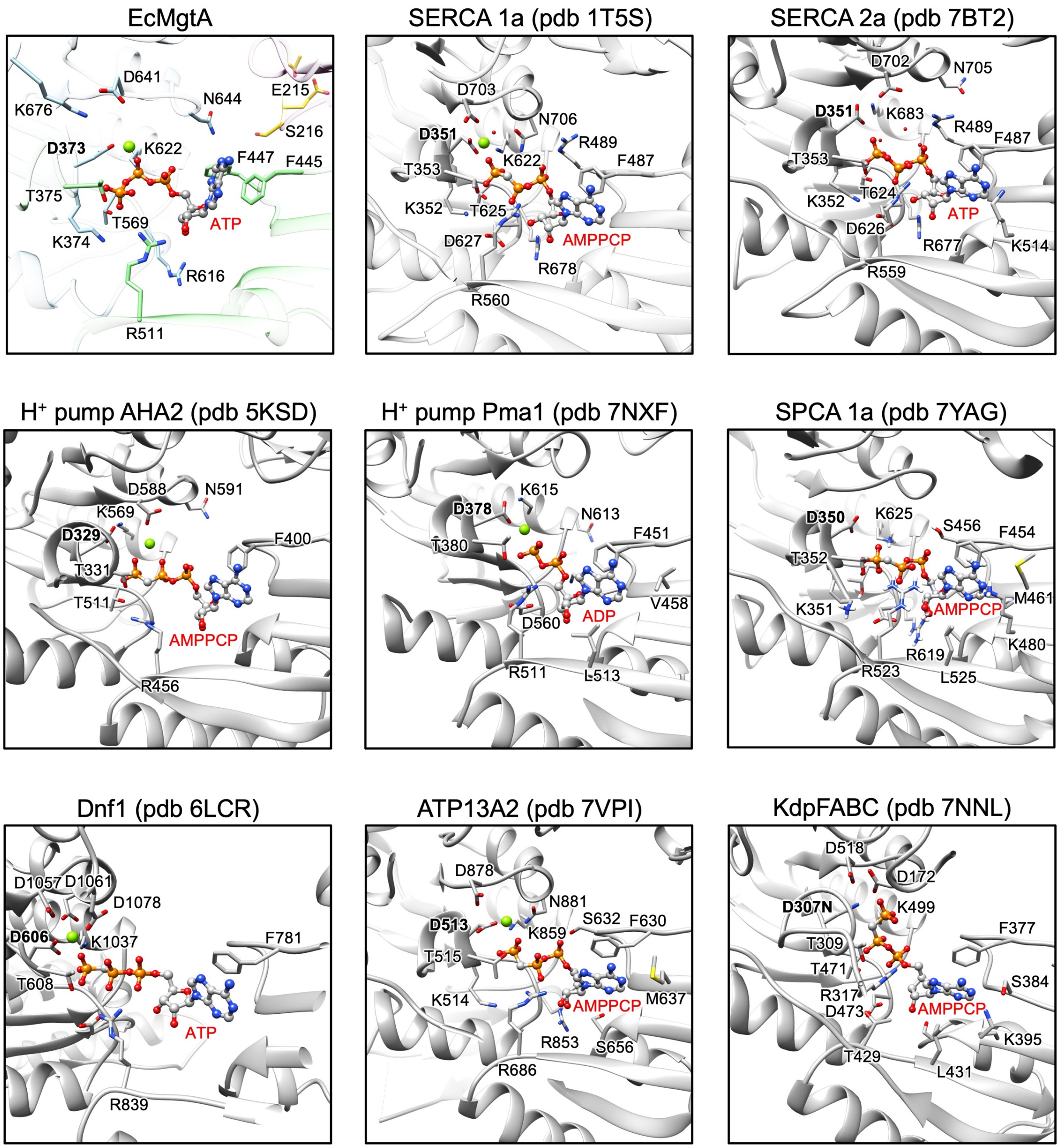

Extended Data Fig 18 The nucleotide binding pocket of MgtA and other P-type ATPases are structurally similar. Zoom in into the nucleotide binding pocket of EcMgtA colored as in Fig 1 and 3. Superimposed nucleotide binding pockets of eight other P-type ATPases are shown in gray. X-ray structure of Ca^{2+} pump SERCA 1a with AMPPCP (PDB 1T5S), X-ray structure SERCA 2a with ATP (PDB 7BT2), X-ray structure of proton pump AHA2 with AMPPCP (PDB 5KSD), X-ray structure of proton pump Pma1 with ADP (PDB 7NXF), Cryo-EM structure of Mn^{2+} and Ca^{2+} pump SPCA with AMPPCP (PDB 7YAG), X-ray structure of phospholipid transporter Dnf1 with ATP (PDB 6LCR), cryo-EM structure of inorganic ion transporter ATP13A2 with AMPPCP (PDB 7VPI), and cryo-EM structure

bioRxiv preprint doi: https://doi.org/10.1101/2024.02.28.582502; this version posted March 1, 2024. The copyright holder for this preprint (which was not certified by peer review) is the author/funder. This article is a US Government work. It is not subject to copyright under 17 USC 105 and is also made available for use under a CC0 license.

of bacterial potassium pump KdpFABC complex with AMPPCP (PDB 7NNL). Residues near the nucleotide binding pocket are displayed as stick, nucleotides as ball-and-stick, Mg^{2+} ions as green spheres.

bioRxiv preprint doi: https://doi.org/10.1101/2024.02.28.582502; this version posted March 1, 2024. The copyright holder for this preprint (which was not certified by peer review) is the author/funder. This article is a US Government work. It is not subject to copyright under 17 USC 105 and is also made available for use under a CC0 license.

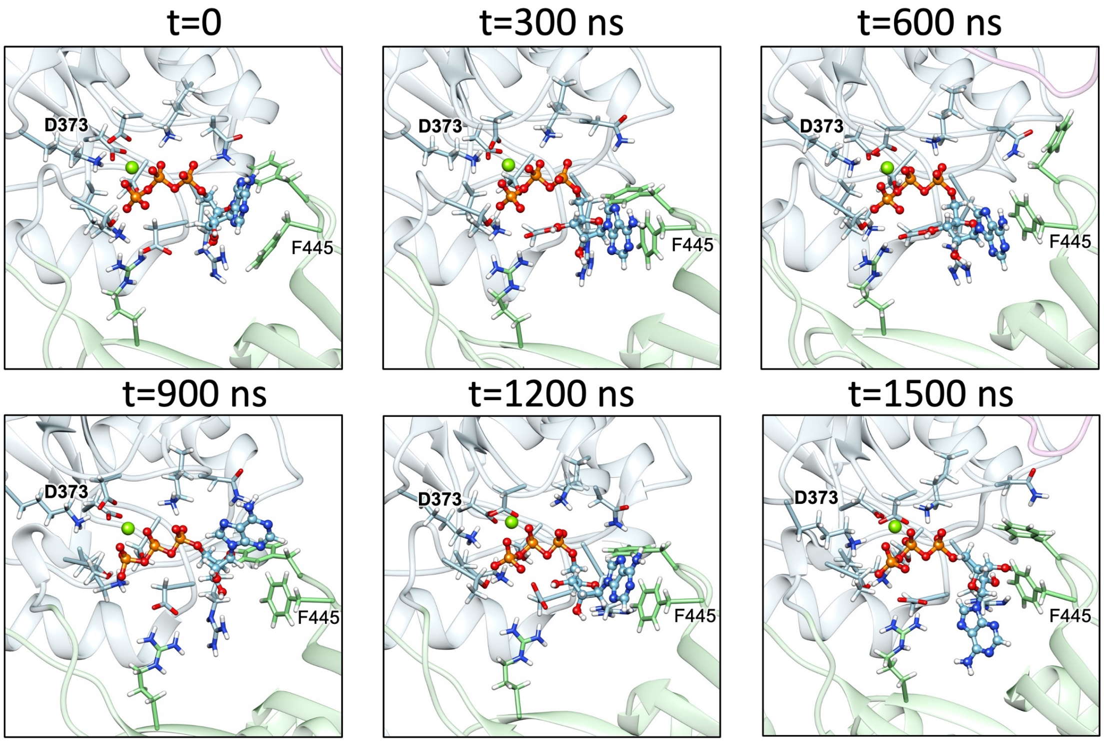

Extended Data Fig 19 Time sequence of MD simulations of the MgtA dimer with ATP. Six representative conformations of the ATP binding pocket during time points in the molecular simulation. The ATP and coordinating residues are displayed according to Extended Data Fig 18.

bioRxiv preprint doi: https://doi.org/10.1101/2024.02.28.582502; this version posted March 1, 2024. The copyright holder for this preprint (which was not certified by peer review) is the author/funder. This article is a US Government work. It is not subject to copyright under 17 USC 105 and is also made available for use under a CC0 license.

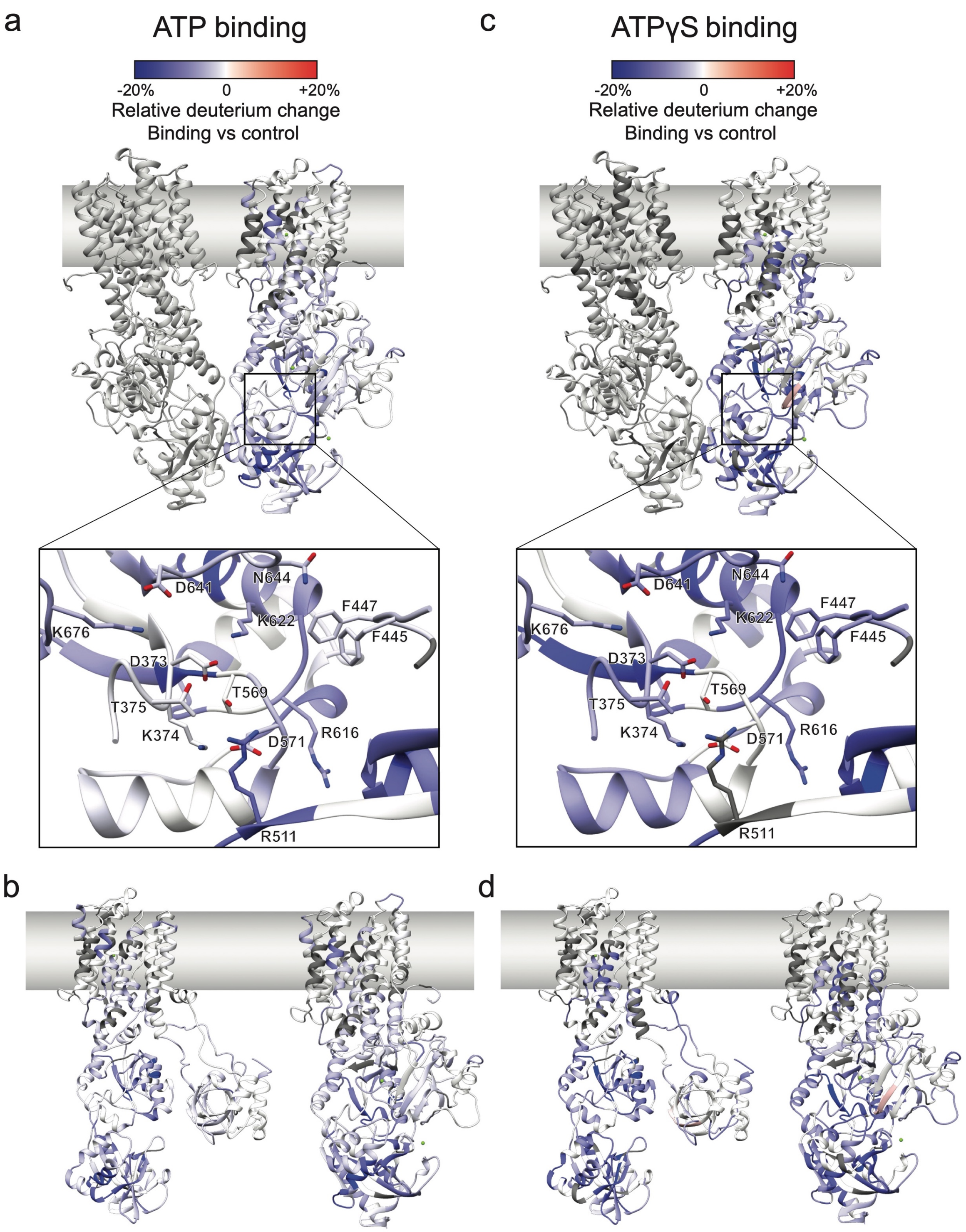

Extended Data Fig 20 HDX-MS analysis of MgtA reveals structural changes upon ATP and ATPγS binding. a, HDX-MS results of 5 mM ATP binding to MgtA after 2 h of deuterium labeling. MgtA is shown as a homodimer in parallel to the membrane in ribbon representation. The ribbon representation has one subunit gray and one subunit of the dimer colored based on uptake differentials. Zoom in for the

bioRxiv preprint doi: https://doi.org/10.1101/2024.02.28.582502; this version posted March 1, 2024. The copyright holder for this preprint (which was not certified by peer review) is the author/funder. This article is a US Government work. It is not subject to copyright under 17 USC 105 and is also made available for use under a CC0 license.

indicated region and display of residues involved in ATP binding. Atoms are colored by heteroatoms and dark gray indicates no peptides were detected. **b**, Comparison of HDX-MS uptake differentials for ATP binding to MgtA mapped onto the monomer and dimer structures. Side view of monomer (left) and one subunit of the dimer (right) MgtA structure colored by HDX-MS uptake differentials between -/+ ATP. **c**, HDX-MS results of 5 mM ATPγS binding to MgtA after 2 h of deuterium labeling. MgtA is shown as a homodimer in parallel to the membrane in ribbon representation. The ribbon representation has one subunit gray and one subunit of the dimer colored based on uptake differentials. Zoom in for the indicated region and display of residues that coordinate ATPγS. Atoms are colored by heteroatoms. **d**, Comparison of HDX-MS uptake differentials for ATPγS binding to MgtA mapped onto the monomer and dimer structures. Side view of monomer (left) and one subunit of the dimer MgtA structure (right) colored by HDX-MS uptake differentials between -/+ ATPγS. For all panels, decreased deuterium uptake is shown in blue, and increased deuterium uptake is shown in red.

bioRxiv preprint doi: https://doi.org/10.1101/2024.02.28.582502; this version posted March 1, 2024. The copyright holder for this preprint (which was not certified by peer review) is the author/funder. This article is a US Government work. It is not subject to copyright under 17 USC 105 and is also made available for use under a CC0 license.

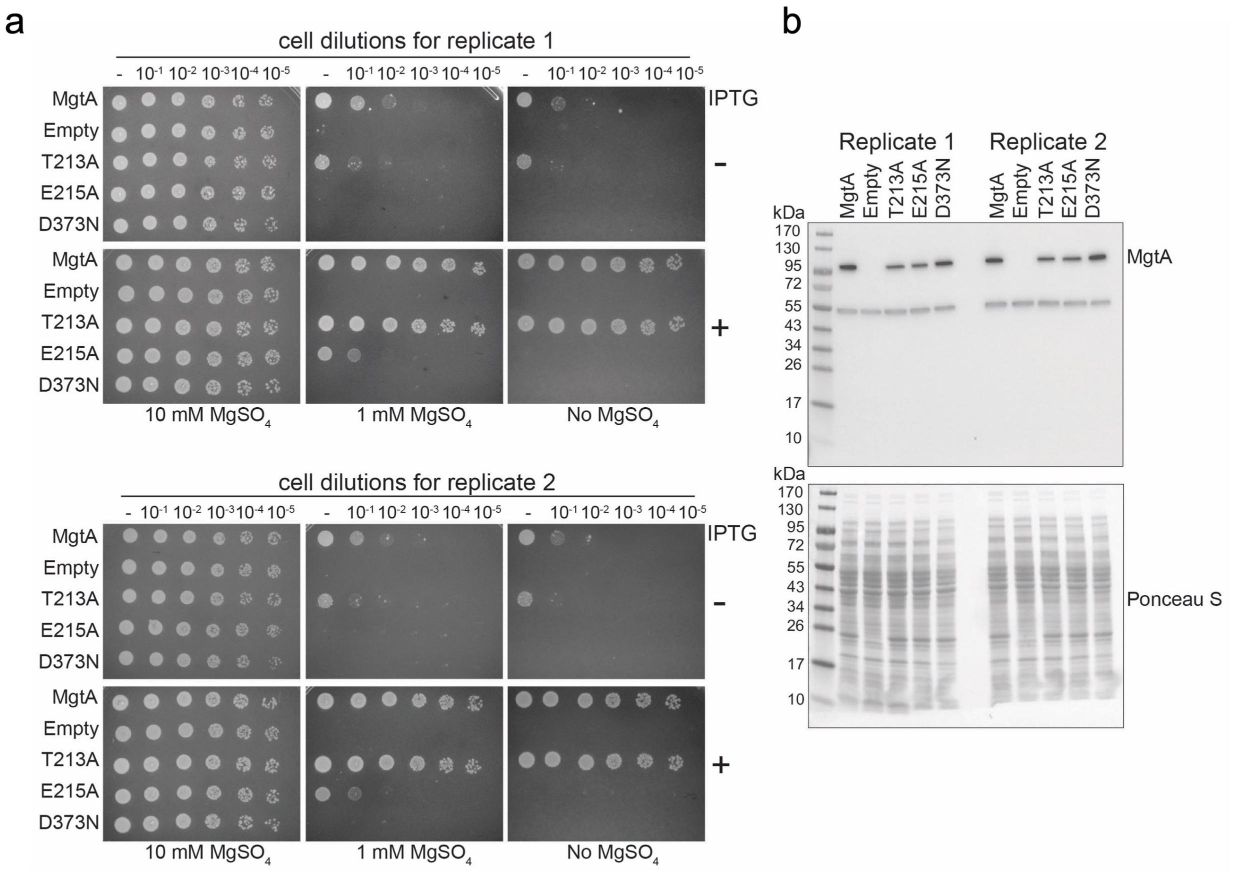

Extended Data Fig. 21 Residues D373 and E215 are required for Mg^{2+} transport. a, $MgtA_{D373N}$ does not complement and $MgtA_{E215A}$ only slightly complements a Mg^{2+}-auxotrophic *E. coli* strain. Overnight cultures were serial diluted and spotted onto LB agar plates supplemented with the indicated concentrations of $MgSO_4$ with (+) and without (-) 0.1 mM IPTG for induction and grown at 37˚C prior to imaging. **b**, MgtA proteins with mutated residues involved in ATP hydrolysis are expressed at levels comparable to the wild-type protein. Cells from the indicated strains were grown uninduced (- IPTG) overnight at 37˚C in LB supplemented with 100 mM $MgSO_4$ and normalized in lysis buffer prior to Western blot analysis with polyclonal anti-MgtA antibodies. Ponceau S-stained membrane serves as a loading control. Results from biological replicates (2n) are shown.

bioRxiv preprint doi: https://doi.org/10.1101/2024.02.28.582502; this version posted March 1, 2024. The copyright holder for this preprint (which was not certified by peer review) is the author/funder. This article is a US Government work. It is not subject to copyright under 17 USC 105 and is also made available for use under a CC0 license.

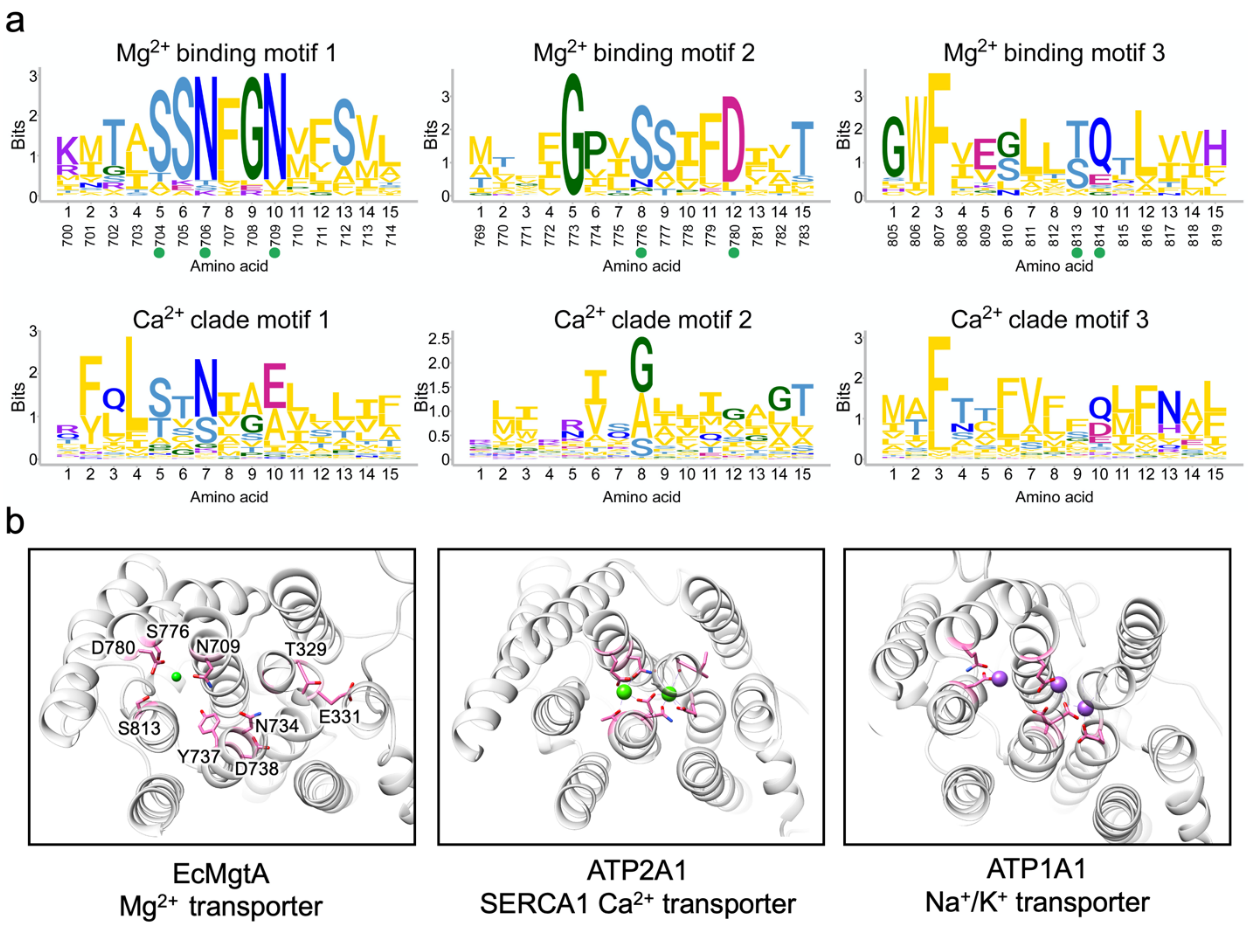

Extended Data Fig. 22 Conservation of key Mg^{2+} binding residues. a, Sequence logos displaying conservation of amino acids in different members of the P-type ATPase transporters. Letters represent amino acid abbreviations; the height of each letter represents the relative probability of conservation among members of the P-type ATPase family. Logos correspond to the Mg^{2+} TM binding sites to illustrate residues conserved in the MgtA clade compared to the Ca^{2+} clade. The sequence logos are also highlighted in the reduced multisequence alignment in Extended Data Fig. 1. **b**, Structural comparison of ion-bound P-type ATPases. One subunit of the dimeric EcMgtA transporter is shown with corresponding regions of ATP2A1 (SERCA1 Ca^{2+} transporter PDB 2ZBD) and ATP1A1 (Na^+/K^+ transporter PDB 4HQJ). Residues of EcMgtA predicted to be involved in ion binding are colored in pink and the Mg^{2+} ion is colored green. Residues of ATP2A1 and ATP1A1 involved in ion binding are colored in pink and the Ca^{2+} and Na^+ ions are colored green and purple, respectively.

bioRxiv preprint doi: https://doi.org/10.1101/2024.02.28.582502; this version posted March 1, 2024. The copyright holder for this preprint (which was not certified by peer review) is the author/funder. This article is a US Government work. It is not subject to copyright under 17 USC 105 and is also made available for use under a CC0 license.

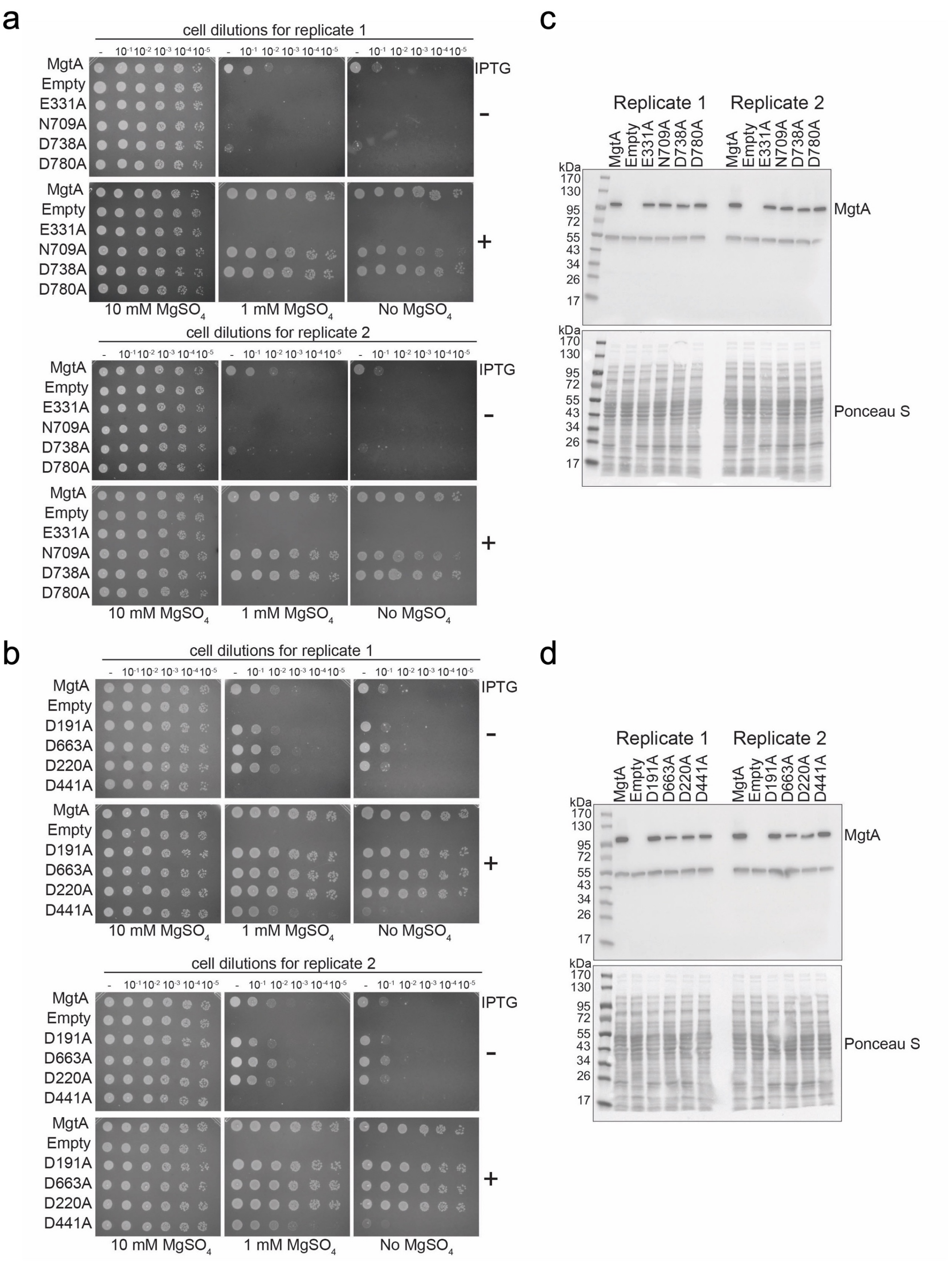

Extended Data Fig. 23 Functional analysis of key Mg^{2+} binding residues. a-b, Complementation assay using a Mg^{2+}-auxotrophic *E. coli* strain and EcMgtA with mutations of Mg^{2+} binding residues contained within TM segments (N709, D780) and based on sequence conservation and structural comparison predicted residues (E331, D738) (**a**) or soluble domain (D191, D663, D220, D441) (**b**). Overnight cultures were serial diluted and spotted onto LB agar plates supplemented with the indicated

bioRxiv preprint doi: https://doi.org/10.1101/2024.02.28.582502; this version posted March 1, 2024. The copyright holder for this preprint (which was not certified by peer review) is the author/funder. This article is a US Government work. It is not subject to copyright under 17 USC 105 and is also made available for use under a CC0 license.

concentrations of $MgSO_4$ with (+) and without (-) 0.1 mM IPTG for induction and grown at 37˚C prior to imaging. **c-d**, Levels of MgtA with mutations of Mg^{2+} binding residues contained within TM segments (E331, N709, D738, D780) (**c**) or soluble domain (D191, D663, D220, D441) (**d**). Cells from the indicated strains were grown uninduced (- IPTG) overnight at 37˚C in LB supplemented with 100 mM $MgSO_4$ and normalized in lysis buffer prior to western blot analysis with polyclonal anti-MgtA antibodies. Ponceau S-stained membrane serves as a loading control. Results from biological replicates (2n) are shown.

bioRxiv preprint doi: https://doi.org/10.1101/2024.02.28.582502; this version posted March 1, 2024. The copyright holder for this preprint (which was not certified by peer review) is the author/funder. This article is a US Government work. It is not subject to copyright under 17 USC 105 and is also made available for use under a CC0 license.

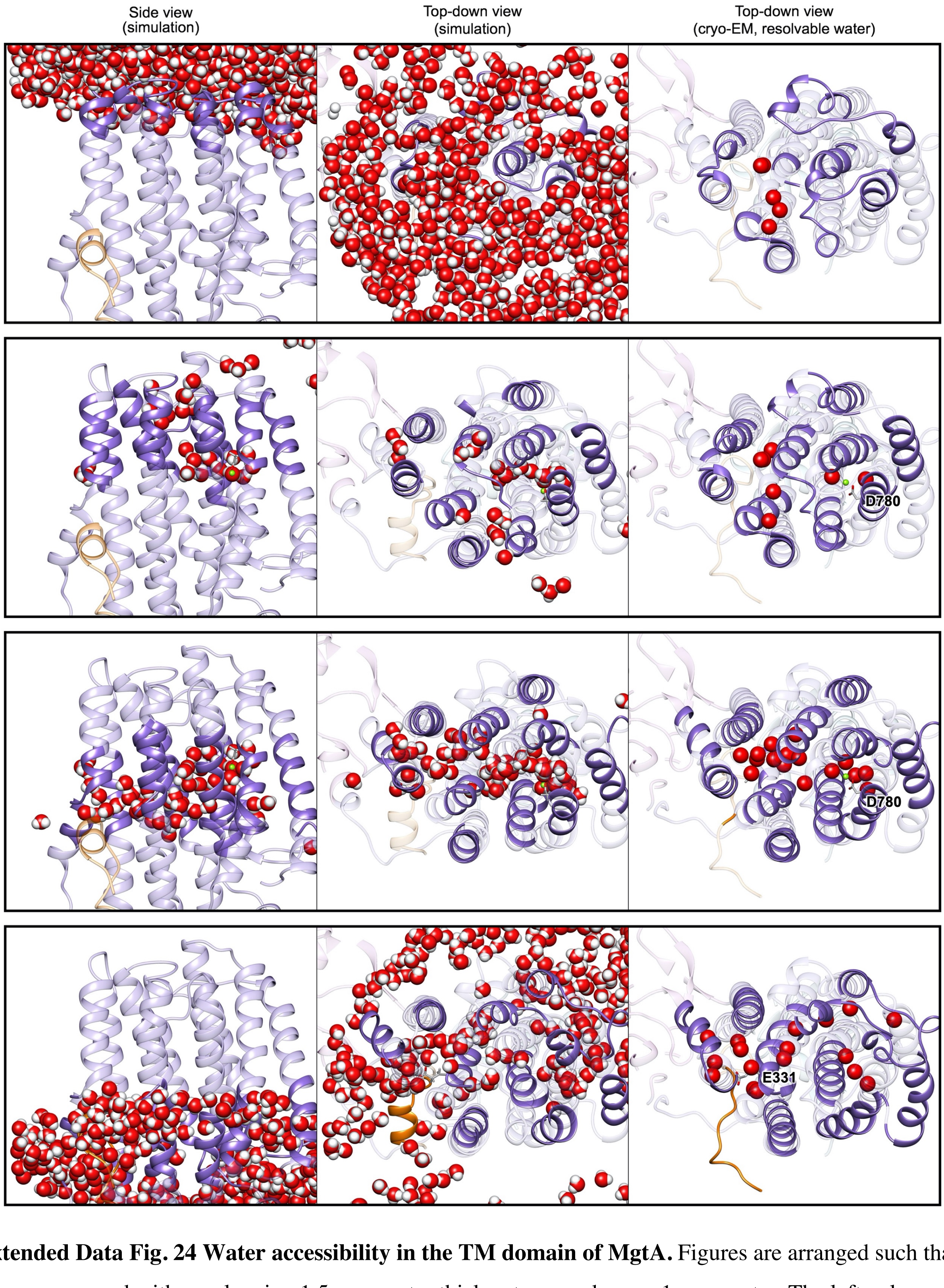

Extended Data Fig. 24 Water accessibility in the TM domain of MgtA. Figures are arranged such that rows correspond with overlapping 1.5 nanometer thick cuts spaced every 1 nanometer. The left column is the side view from the simulation, while the middle column is from the top-down starting on the

bioRxiv preprint doi: https://doi.org/10.1101/2024.02.28.582502; this version posted March 1, 2024. The copyright holder for this preprint (which was not certified by peer review) is the author/funder. This article is a US Government work. It is not subject to copyright under 17 USC 105 and is also made available for use under a CC0 license.

periplasmic side of the transporter. For the simulation, waters (including hydrogens) are shown in sphere representation. At right is the corresponding top-down view of the MgtA dimer from cryo-EM, with resolved water molecules shown as red spheres. The protein ribbon model is shown opaque through the cut of the simulation, while outside of the cut waters are not shown, and the protein is transparent. The transmembrane Mg^{2+} as well as glutamic and aspartic acid residues 331 and 780 (respectively) are shown in stick representation.

bioRxiv preprint doi: https://doi.org/10.1101/2024.02.28.582502; this version posted March 1, 2024. The copyright holder for this preprint (which was not certified by peer review) is the author/funder. This article is a US Government work. It is not subject to copyright under 17 USC 105 and is also made available for use under a CC0 license.

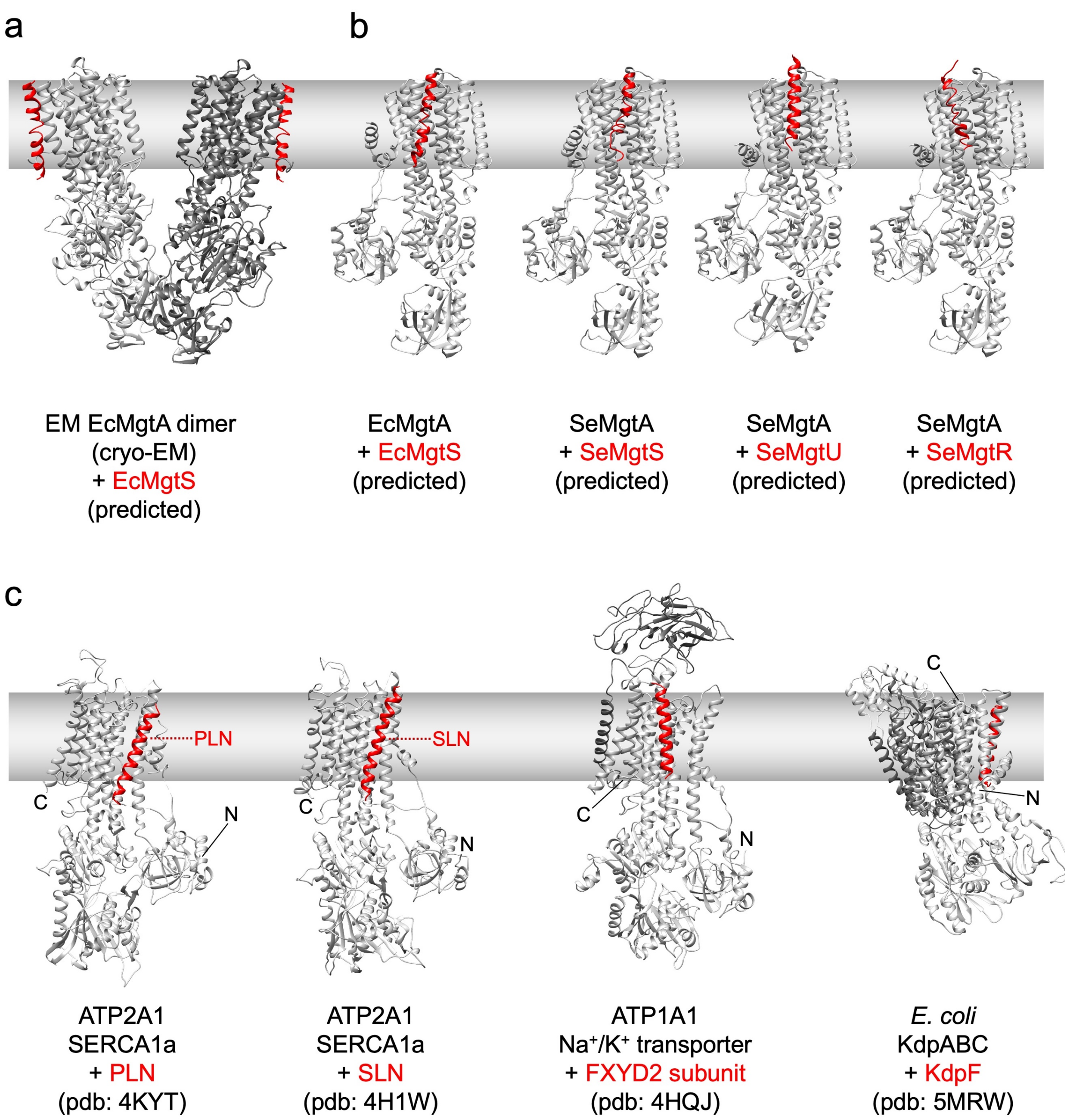

Extended Data Fig. 25 Predicted and documented interactions of small proteins with P-type ATPase proteins. a, Dimer cryo-EM structure of *E. coli* MgtA from this study with *E. coli* MgtS binding predicted by AlphaFold Multimer beta. **b**, Models of *E. coli* MgtA monomer and *E. coli* MgtS and *S. enterica* MgtA and *S. enterica* MgtS, MgtU and MgtR predicted by AlphaFold Multimer beta. **c**, Selected structures of indicated P-type ATPases solved with small α-helical proteins. P-type ATPases are in gray with small protein in red.

bioRxiv preprint doi: https://doi.org/10.1101/2024.02.28.582502; this version posted March 1, 2024. The copyright holder for this preprint (which was not certified by peer review) is the author/funder. This article is a US Government work. It is not subject to copyright under 17 USC 105 and is also made available for use under a CC0 license.

Extended Data Tables

Extended Data Table 1. Residues involved in dimer interface.

Residue chain A	Residue chain B	Interaction Type
Q380	Q380	Electrostatic/h-bond
K382	E582	Electrostatic
N387	Q508	Electrostatic/h-bond
K548	E549	Electrostatic
E386	R578	Electrostatic<br>(in simulation)
V384	L544	Hydrophobic
V384	P546	Hydrophobic
V384	P547	Hydrophobic
L385	L385	Hydrophobic
L385	L510	Hydrophobic
L385	L544	Hydrophobic
L544	L544	Hydrophobic
P546	P546	Hydrophobic

bioRxiv preprint doi: https://doi.org/10.1101/2024.02.28.582502; this version posted March 1, 2024. The copyright holder for this preprint (which was not certified by peer review) is the author/funder. This article is a US Government work. It is not subject to copyright under 17 USC 105 and is also made available for use under a CC0 license.

Extended Data Table 2. Summary of all mutants generated and their outcome.

Mutation	Functional Significance	Outcome
D373N	DxT motif, ATP hydrolysis	No Activity
T213A	TGES loop, ATP hydrolysis	Reduced activity mild
E215A	TGES loop, ATP hydrolysis	Reduced activity severe
E331A	TM Mg^{2+} binding site	No activity
D738A	TM Mg^{2+} binding site	Reduced activity mild
D780A	TM Mg^{2+} binding site	No activity
N709A	TM Mg^{2+} binding site	Reduced activity mild
D191A	Site II cytoplasmic Mg^{2+} binding	Active
D663A	Site II cytoplasmic Mg^{2+} binding	Active
E220A	Site III cytoplasmic Mg^{2+} binding	Active
D441A	Site III cytoplasmic Mg^{2+} binding	Reduced activity severe
Q380A, V384A, L385A, L544K, E549K	Dimer interface	Reduced activity mild
Q380A, K382A, V384A, L385A, L544K, E549K	Dimer interface	Reduced activity mild
Q380A, K382A, V384A, L385A, L544K, E549K, E582K	Dimer interface	Reduced activity mild
Q380A, K382A, V384A, L385A, N387G, Q508A, L510A, L544S, K548A, E549A, T550A	Dimer interface	No activity
Δ36	N-terminus	Unstable
Δ54	N-terminus	Unstable
K3A, E4A, F6A	N-terminus	Unstable

bioRxiv preprint doi: https://doi.org/10.1101/2024.02.28.582502; this version posted March 1, 2024. The copyright holder for this preprint (which was not certified by peer review) is the author/funder. This article is a US Government work. It is not subject to copyright under 17 USC 105 and is also made available for use under a CC0 license.

Extended Data Table 3. Cryo-EM data collection parameters and analysis.

	**Dataset 1**	**Dataset 2**	**Dataset 3**	**Dataset 4**	**Dataset 5**	**Dataset 6**	**Dataset 7**
**Date**	2021/09/16-19	2021/10/29-31	2022/08/01-02	2022/08/23-25	2022/08/03-04	2022/10/28-30	2022/09/26-29
**Protein concentration**	3.2 mg/mL	3.2 mg/mL	2.3 mg/mL	2.3 mg/mL	2.3 mg/mL	2.3 mg/mL	2.3 mg/mL
**Ligands**	(Mg)	(Mg)	Mg-ATPγS	Mg-ATPγS	Mg-ADP	Mg-ADP	Mg-ATP
**Sample volume**	3 μl	3 μl	3 μl	3 μl	3 μl	3 μl	3 μl
**Grid type**	QF R1.2/1.3 400 Cu mesh	QF R1.2/1.3 400 Cu mesh	QF R1.2/1.3 400 Cu mesh	QF R1.2/1.3 400 Cu mesh	QF R1.2/1.3 400 Cu mesh	QF R1.2/1.3 400 Cu mesh	QF R1.2/1.3 400 Cu mesh
**Plunge freezer**	Leica EM GP2	Leica EM GP2	Leica EM GP2	Leica EM GP2	Leica EM GP2	Leica EM GP2	Leica EM GP2
**Blotting time (s)**	5	4	6	6	6	6	6
**Temperature (°C)**	5	5	4	4	4	4	4
**humidity set (measured)**	95% (77-88%)	95% (77-88%)	95% (77-88%)	95% (77-88%)	95% (77-88%)	95% (77-88%)	95% (77-88%)
**Microscope**	Titan Krios G1	Titan Krios G1	Titan Krios G1	Titan Krios G1	Titan Krios G1	Titan Krios G1	Titan Krios G4
**Voltage (kV)**	300	300	300	300	300	300	300
**Camera**	K3 (CDS mode)	K3 (CDS mode)	K3 (CDS mode)	K3 (CDS mode)	K3 (CDS mode)	K3 (CDS mode)	K3 (CDS mode)
**Energy filter (slit)**	Yes (20 eV)	Yes (20 eV)	Yes (20 eV)	Yes (20 eV)	Yes (20 eV)	Yes (20 eV)	Yes (20 eV)
**Cs corrector**	No	No	No	No	No	No	No
**Objective aperature**	C2 100 μm	C2 70 μm	C2 100 μm	C2 100 μm	C2 100 μm	C2 100 μm	No
**Magnification**	105,000 x	105,000 x	105,000 x	105,000 x	105,000 x	105,000 x	105,000 x
**Physical pixel size (Å/px)**	0.83	0.83	0.83	0.83	0.83	0.83	0.85
**Super-resolution pixel size (Å/px)**	0.415	0.415	0.415	0.415	0.415	0.415	0.425
**Electron exposure (e^-/Å^2)**	60	50	50	50	50	50	50
**Number of movie frames**	60	50	50	50	50	50	60
**Dose rate (e^-/px/s)**	9.5 (~7.5 on camera)	10 (~7.5 on camera)	9 (~7.5 on camera)	9 (~7.5 on camera)	9 (~7.5 on camera)	9 (~7.5 on camera)	9 (~7.5 on camera)
**Defocus (μm)**	-1 to -2	-1 to -2	-0.7 to -1.8	-0.7 to -1.8	-0.8 to -1.5	-0.8 to -1.5	-0.8 to -1.8
**Number of total micrographs**	10,246	6,906	3,396	6,472	2,519	7,107	14,585
**Number of selected micrographs**	10,164	5,778	2,849	6,337	1,897	7,082	11,446
**Number of particles picked**	1,578,214	1,775,726	454,311	1,329,014	302,981	1,153,466	2,339,956
**Number of MgtA dimer particles**	497,633	149,351	22,495	43,993	16,962	48,407	94,029
**Number of MgtA monomer particles**	609,684	323,129	N/A	N/A	N/A	N/A	N/A

bioRxiv preprint doi: https://doi.org/10.1101/2024.02.28.582502; this version posted March 1, 2024. The copyright holder for this preprint (which was not certified by peer review) is the author/funder. This article is a US Government work. It is not subject to copyright under 17 USC 105 and is also made available for use under a CC0 license.

Extended Data Table 4. Cryo-EM map and model analysis.

	MgtA dimer<br><br>(C2)<br>EMD-42794<br>PDB 8UY7	MgtA dimer<br><br>(C1)<br>EMD-42795<br>PDB 8UY8	MgtA monomer<br><br>(C1)<br>EMD-42796<br>PDB 8UY9	MgtA dimer<br>with ATPγS<br>(C2)<br>EMD-42798<br>PDB 8UYB	MgtA dimer<br>with ADP<br>(C2)<br>EMD-42799<br>PDB 8UYC	MgtA dimer<br>with ATP<br>(C2)<br>EMD-42797<br>PDB 8UYA
**Number of particles in map**	160,139	160,139	78,231	28,120	28,666	48,080
**Final pixel size used for final maps (Å/px)**	0.83	0.83	0.83	0.83	0.83	0.85
**Resolution of map (Å)**	2.93	3.03	3.65	3.87	3.75	3.72
**Local resolution range (Å)**	1.9 -3.3	2.0 - 3.7	2.3 - 8.0	2.5 – 5.0	2.5 – 4.5	2.5 – 4.0
**B-factor for map ($Å^2$)**	104	92	106	123	110	133
**Non-hydrogen atoms**	14,038	13,930	6,587	13,882	13,828	13,904
**Residues**	1,796	1794	859	1,796	1,796	1,796
**Ligands**	6	6	1	8	6	10
**Bond lengths (Å)**	0.003	0.003	0.003	0.002	0.002	0.002
**Bond angle (°)**	0.612	0.551	0.652	0.511	0.517	0.54
**MolProbity score**	1.62	1.64	1.64	1.75	1.64	1.82
**Clashscore**	7.49	6.73	9.69	8.53	7.64	6.44
**Rotamer outliers (%)**	0.06	0.00	0.00	0.00	0.00	0.00
**Ramachandran favored (%)**	96.65	96.20	97.32	95.81	96.54	92.63
**Ramachandran allowed (%)**	3.29	3.74	2.68	4.19	3.46	7.37
**Ramachandran outliers (%)**	0.06	0.06	0.00	0.00	0.00	0.00
**Number of water molecules**	200	150	0	0	0	14
**Number of Mg^{2+} ions**	6	6	1	6	6	8
**Number of nucleotides**	0	0	0	2	0	2

Additional Data

Supplementary Data 1 Extended multisequence alignment of all P-type ATPases (see separate .aln file).

Supplementary Data 2 Primers, plasmids, and strains used in this study (see separate .xlsx file).

bioRxiv preprint doi: https://doi.org/10.1101/2024.02.28.582502; this version posted March 1, 2024. The copyright holder for this preprint (which was not certified by peer review) is the author/funder. This article is a US Government work. It is not subject to copyright under 17 USC 105 and is also made available for use under a CC0 license.

Extended Data Movies

Extended Data Movie 1. 360 degree view of the dimeric cryo-EM map of *E. coli* Mg^{2+} transporter MgtA.

Extended Data Movie 2. 360 degree view of the monomeric cryo-EM map of *E. coli* Mg^{2+} transporter MgtA.

Extended Data Movie 3. Morph between the structural model of a single subunit of the dimeric and monomeric *E. coli* Mg^{2+} transporter MgtA.

Extended Data Movie 4. MD simulation movie of the N-terminal tail of the *E. coli* Mg^{2+} transporter MgtA.

Extended Data Movie 5. MD simulation movie of the *E. coli* Mg^{2+} transporter MgtA showing the full dimer (in color) aligned to the cryo-EM structure (grey).

Extended Data Movie 6. Zoomed in MD simulation movie of the *E. coli* Mg^{2+} transporter MgtA dimer interface.

Extended Data Movie 7. The *E. coli* Mg^{2+} transporter MgtA dimer with ATP bound.

Extended Data Movie 8. MD simulation movie of the Mg^{2+} ion in the middle of the transmembrane domains of the *E. coli* Mg^{2+} transporter MgtA.
